# Reporting Methods of Blinding in Randomized Trials Assessing Nonpharmacological Treatments 

**DOI:** 10.1371/journal.pmed.0040061

**Published:** 2007-02-20

**Authors:** Isabelle Boutron, Lydia Guittet, Candice Estellat, David Moher, Asbjørn Hróbjartsson, Philippe Ravaud

**Affiliations:** 1 INSERM, U738, Paris, France; 2 Université Paris 7, UFR de Médecine, Paris, France; 3 AP-HP, Hôpital Bichat, Département d'Epidémiologie, Biostastique et Recherche Clinique, Paris, France; 4 Children's Hospital of Eastern Ontario Research Institute, University of Ottawa, Ottawa, Canada; 5 Department of Epidemiology and Community Medicine, Faculty of Medicine, University of Ottawa, Ottawa, Canada; 6 Nordic Cochrane Centre, Rigshospitalet, Copenhagen, Denmark; University of Glasgow, United Kingdom

## Abstract

**Background:**

Blinding is a cornerstone of treatment evaluation. Blinding is more difficult to obtain in trials assessing nonpharmacological treatment and frequently relies on “creative” (nonstandard) methods. The purpose of this study was to systematically describe the strategies used to obtain blinding in a sample of randomized controlled trials of nonpharmacological treatment.

**Methods and Findings:**

We systematically searched in Medline and the Cochrane Methodology Register for randomized controlled trials (RCTs) assessing nonpharmacological treatment with blinding, published during 2004 in high-impact-factor journals. Data were extracted using a standardized extraction form. We identified 145 articles, with the method of blinding described in 123 of the reports. Methods of blinding of participants and/or health care providers and/or other caregivers concerned mainly use of sham procedures such as simulation of surgical procedures, similar attention-control interventions, or a placebo with a different mode of administration for rehabilitation or psychotherapy. Trials assessing devices reported various placebo interventions such as use of sham prosthesis, identical apparatus (e.g., identical but inactivated machine or use of activated machine with a barrier to block the treatment), or simulation of using a device. Blinding participants to the study hypothesis was also an important method of blinding. The methods reported for blinding outcome assessors relied mainly on centralized assessment of paraclinical examinations, clinical examinations (i.e., use of video, audiotape, photography), or adjudications of clinical events.

**Conclusions:**

This study classifies blinding methods and provides a detailed description of methods that could overcome some barriers of blinding in clinical trials assessing nonpharmacological treatment, and provides information for readers assessing the quality of results of such trials.

## Abstract 2

An assessment of blinding methods used in nonpharmacological trials published in one year in high-impact factor journals classifies methods used and describes methods that could overcome some barriers of blinding.

## Editors' Summary

**Background.:**

Well-conducted “randomized controlled trials” are generally considered to be the most reliable source of information about the effects of medical treatments. In a randomized trial, the play of chance is used to decide whether each patient receives the treatment under investigation, or whether he/she is assigned to a “control” group receiving the standard treatment for their condition. This helps makes sure that the two groups of patients receiving the different treatments are equivalent at the start of the trial. Proper randomization also prevents doctors from deciding which treatment individual patients are given, which could distort the results. An additional technique used is “blinding,” which involves taking steps to prevent patients, doctors, or other people involved in the trial (e.g., those recording measurements) from finding out which patients have received which treatment. Properly done, blinding should make sure the results of a trial are more accurate. This is because in an unblinded study, participants may respond better if they know they have received a promising new treatment (or worse if they only got a placebo or an old drug). In addition, doctors and others in the research team may “want” a particular treatment to perform better in the trial, and unthinking bias could creep into their measurements or actions. However, blinding is not a simple, single step; the people carrying out the trial often have to set up a variety of different procedures.

**Why Was This Study Done?:**

The authors of this study had already conducted research into the way in which blinding is done in trials involving drug (“pharmacological”) treatment. Their work was published in October 2006 in *PLoS Medicine*. However, concealing from patients the type of pill that they are being given is much easier than, for example, concealing whether or not they are having surgery or whether or not they are having psychotherapy. The authors therefore set out to look at the methods that are in use for blinding in nonpharmacological trials. They hoped that a better understanding of the different blinding methods would help people doing trials to design better trials in the future, and also help readers to interpret the quality of completed trials.

**What Did the Researchers Do and Find?:**

The authors systematically searched the published medical literature to find all randomized, blinded drug trials published in just one year (2004) in a number of different “high-impact” journals (well-regarded journals whose articles are often mentioned in other articles). Then, they classified information from the published trial reports. They ended up with 145 trial reports, of which 123 described how blinding was done. The trials covered a wide range of medical conditions and types of treatment. The blinding methods used mainly involved the use of “sham” procedures. Thus, in 80% of the studies in which the treatment involved a medical device, a pretend device had been used to make patients in the control group think they were receiving treatment. In many of the treatments involving surgery, researchers had devised elaborate ways of making patients think they had had an operation. When the treatment involved manipulation (e.g. physiotherapy or chiropractic), fake “hands-on” techniques were given to the control patients. The authors of this systematic review classify all the other techniques that were used to blind both the patients and members of the research teams. They found that some highly innovative ideas have been successfully put into practice.

**What Do These Findings Mean?:**

The authors have provided a detailed description of methods that could overcome some barriers of blinding in clinical trials assessing nonpharmacological treatment. The classification of the techniques used will be useful for other researchers considering what sort of blinding they will use in their own research.

**Additional Information.:**

Please access these Web sites via the online version of this summary at http://dx.doi.org/10.1371/journal.pmed.0040061.

- The James Lind Library has been created to help patients and researchers understand fair tests of treatments in health care by illustrating how fair tests have developed over the centuries
- ClinicalTrials.gov, a trial registry created by the US National Institutes of Health, has an introduction to understanding clinical trials
- The UK National Health Service National Electronic Library for Health has an introduction to controlled clinical trials
- The CONSORT statement is intended to strengthen evidence-based reporting of clinical trials

## Introduction

Bias in clinical research can be described as systematic error that can result in false treatment effect estimates [1]. Bias may cause a investigators to misinterpret the result of any research finding. Randomized controlled trials (RCTs) are widely recognized as being the best design for avoiding and/or minimizing bias. Blinding, although not possible in every study, is an important methodological technique to help reduce the influence bias may have on the results of an evaluation [2–9]. Blinding refers to keeping key persons, such as participants, health care providers, and outcome assessors, unaware of the treatment administered or of the true hypothesis of the trial [3,5,6]. Blinding of participants and health care providers prevents performance bias that occurs if additional therapeutic interventions (i.e., cointerventions) are provided preferentially in one of the comparison groups [10]. Blinding of outcome assessors minimizes the risk of detection bias (i.e., observer, ascertainment, assessment bias). This type of bias occurs if participant assignment influences the process of outcome assessment [10]. For example, nonblinded neurologists assessing the outcome from a trial [9] demonstrated an apparent treatment benefit, whereas blinded neurologists did not. Finally, blinding of data analysts can also prevent bias, as knowledge of the intervention received may influence the choice of analytical strategies and methods [10]. Empirical evidence has demonstrated that lack of reporting of double blinding is responsible for biased treatment estimates [1,2,11–13].

However, blinding might be difficult or impossible to establish and/or to maintain [14–16], and a blinding procedure might fail [17,18]. Therefore, researchers and readers must be aware of existing methods of blinding to be able to appraise the feasibility of blinding in a trial.

Nonpharmacological treatment (NPT) such as surgery, technical interventions, rehabilitation, behavioral interventions, psychotherapy, and use of devices represent a wide range of treatment options. Assessing NPT raises specific methodological issues [19–26]. Blinding is less frequently reported in RCTs assessing NPT [19], possibly due to the difficulty in achieving and maintaining it [18] and a lack of knowledge about existing methods in the field. Here, we present an inventory and classification scheme of reported methods of blinding procedures used in NPT trials.

## Methods

### Domain of Interest and Defining NPT

We assessed the reporting of blinding and the method of blinding participants, health care providers (i.e., those administering the treatment), and outcome assessors (i.e., those assessing the main outcome). Our assessment included the blinding status of other caregivers such as physicians administering cointerventions because they may have an important influence on treatment effect in trials assessing NPT. For example, in a trial assessing a surgical procedure, even if the surgeon could not be blinded, it is possible that the health care professionals following participants after the procedure could be blinded, and contact between other caregivers and the surgeon could be avoided. We did not focus on other key trial participants such as data analysts, because there is no barrier for blinding data analysts in trials assessing NPT. Furthermore, we did not provide data on the methods of blinding data collectors, because this information is usually poorly reported, and consistently identifying data collectors in articles is difficult [27].

NPT is defined as any intervention(s) provided to participants that involves treatments other than the use of pharmaceuticals. These treatments include, for example, surgery, technical operations (e.g., angioplasty, joint lavage), implanted devices (pacemaker, stent, ventriculoperitoneal shunt, ear tube, or prosthetic device), nonimplantable devices, rehabilitation, behavioral therapy, and psychotherapy but not the organization of the health care system or interventions provided to health care providers.

### Identification of Reports

We identified reports of all RCTs published in 2004 in the ten highest impact factor general and internal medicine journals and the three highest impact factors for each subject category of the Journal Citation Reports (Journal citation reports, http://isiwebofknowledge.com) such as cardiac and cardiovascular system, respiratory system, or rheumatology and indexed in Medline by searching PubMed (http://www.ncbi.nlm.nih.gov/entrez/query.fcgi) using the terms “double-blind method” OR “single-blind method,” limited to RCTs (Text S1). We chose these journals because a high impact factor is a good predictor of high methodological quality of journal articles [28]. We also searched for reports on blinding indexed in the Cochrane Methodology Register (Cochrane Library, Issue 1, 2005, http://www3.cochrane.org/access_data/cmr/accessDB_cmr.asp) using the term “blind,” with no limitation linked to the year of publication but a language limitation (English or French).

### Eligibility Criteria and Screening

We collected records in an EndNote data file, and one of us (IB) assessed each one by screening the titles and abstracts to identify the relevant studies. Full articles were retrieved for further assessment if the information given suggested that the study was a randomized controlled trial assessing NPT. If there was any doubt regarding these criteria from the information given in the title and abstract, the full article was retrieved for clarification.

After obtaining the full text, we further selected reports only if they reported that participants, health care providers, other caregivers, or outcome assessors were blinded. We excluded nonrandomized trials, extended follow-up trials (i.e., extended follow-up of patients included in an RCT beyond the last outcome assessment), diagnostic accuracy studies, pathophysiological studies, an RCT ancillary study (such as a subgroup analysis, cost-effectiveness evaluations, systematic reviews and/or meta-analyses), and any trials assessing pharmacological treatment. However, trials comparing nonpharmacological and pharmacological treatments were selected.

Additional relevant trials were found by searching reference lists of relevant selected reports or those that were known by members of our team or experts in this field, with no limitation linked to the year of publication.

### Data Collection

From a review of the relevant literature, we generated a standardized data collection form that was iterated among the research team. Before data extraction, as a calibration exercise, two members of the team (IB, LG) independently evaluated a separate set of ten reports. A meeting followed in which the ratings were reviewed and any disagreements were resolved by consensus. One reviewer (IB) independently completed all the data extractions. A second member of the team reviewed a random sample of 30 articles as a quality assurance exercise. The reproducibility was good, with the rate of agreement higher than 85% for the main items.

The form included 46 items (available upon request) that described the experimental treatment (nonimplantable devices, surgery and technical operation, implantable devices, rehabilitation, education, diet, psychotherapy) and the control treatment (placebo, active control treatment, usual care). We classified the NPT using three categories based on our experience, according to the difficulties of blinding: (1) surgery or technical procedures, (2) participative interventions such as rehabilitation, education, or psychotherapy involving a collaboration between participants and care providers, and (3) devices.

We identified and classified the primary outcome as a participant-reported outcome according to specific criteria [18]: the participant as the outcome assessor (e.g., pain, disability, quality of life), physician-driven data that assume contact between participants and outcome assessors (e.g., range of motion), complementary investigations that do not assume contact between participants and outcome assessors (e.g., international normalized ratios within the target range, angiographic restenosis) or a clinical event determined by the interaction between participants and care providers (e.g., death, myocardial infarction, stroke, dialysis, blood transfusion).

We extracted the reported blinding status for participants, health care providers, other caregivers, and outcome assessors and checked whether the blinding method(s) were described and, if so, how. Each report was reviewed for any description of a sham procedure; an attempt to blind participants, health care providers, or outcome assessors to the hypothesis; or an attempt to blind other caregivers who did not perform the treatment but administered cointerventions. Finally, we recorded the description of specific methods of blinded outcome assessments, such as participants being informed not to reveal to the outcome assessors what intervention they received and whether the success of blinding was tested.

### Classification of the Methods of Blinding

We classified how blinding was achieved for participants, health care providers, and the main outcome assessor mainly on the basis of what seemed reasonable after having read through the various methods of blinding.

### Statistical Analysis

Descriptive statistics for categorical variables were described with frequencies and percentages. All data analyses involved SAS for Windows, Release 9.1 (http://www.sas.com).

## Results

### Articles Selected

**Figure 1:**
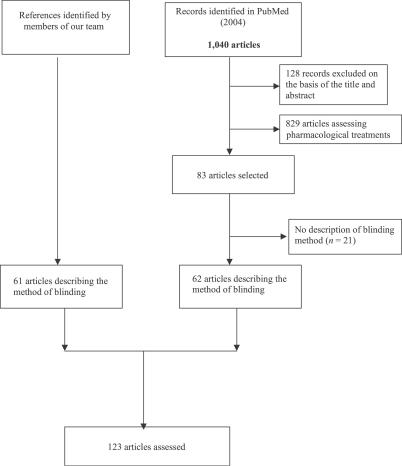
Flow of Records, Citations, and Articles through the Cross-Sectional Study

Our electronic search retrieved 1,040 records. We identified 83 reports on the basis of the title and abstract and 61 articles by personal identification and use of references. Finally, we analyzed 123 reports describing a blinding method (Figure 1).

### Characteristics of the Included Articles

Table 1 describes the characteristics of the included articles. These reports assessed devices (*n* = 59; 48%), participative interventions (i.e., interventions involving a collaboration between participants and care providers) such as rehabilitation, education, psychotherapy (*n* = 33; 27%), surgery or technical interventions (*n* = 20; 16%), or other NPT such as preoperative autologous blood donation, hyperbaric oxygen therapy, or blood patch (*n* = 11; 9%). A total of 59% of reports (*n* = 73) clearly described blinding for participants, 31% (*n* = 39) health care providers or other caregivers, and 86% (*n* = 105) outcome assessors. Twenty-one reports (17%) assessed the success of blinding.

**Table 1:**
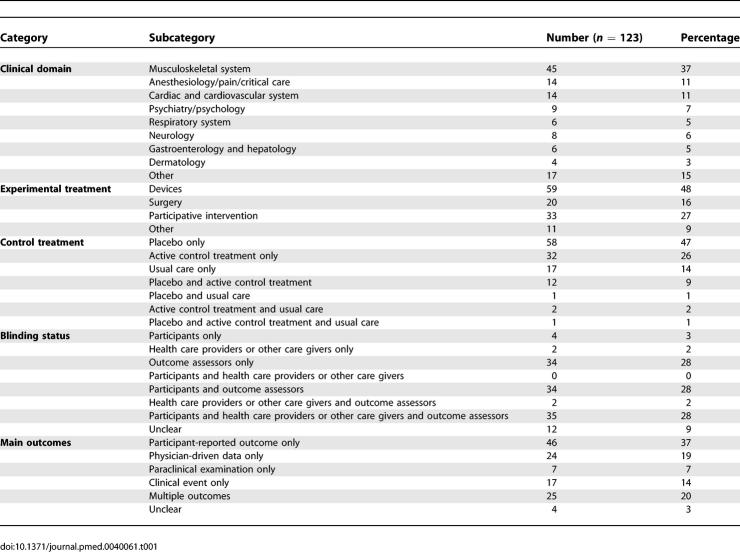
Characteristics of the Included Articles to Assess Blinding in Reports of Trials Investigating Nonpharmacologic Treatment

### Method of Blinding

#### Use of sham procedures.

**Figure 2:**
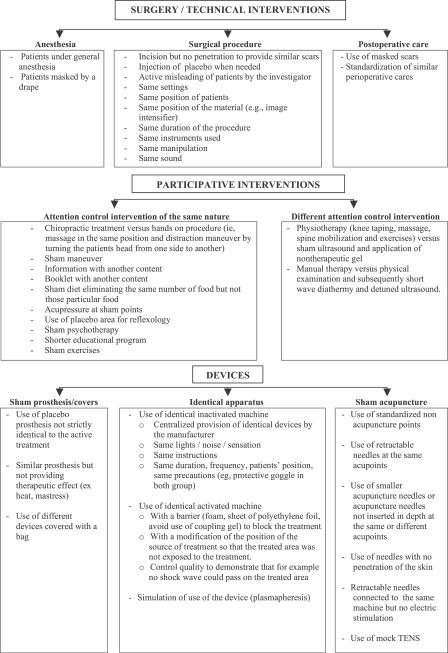
Sham Procedure Performed According to the Category of Treatment Assessed

Almost half the reports (58%; *n* = 71) described the use of a sham procedure, 80% of the 59 reports assessing a device, 45% of the 20 reports assessing a surgical procedure, 36% of the 33 reports assessing participative interventions and 27% of the 11 reports on other NPT. Figure 2 describes these sham procedures according to the treatment assessed. Table S1 provides examples of reporting of sham procedures.

For surgical interventions, the sham procedures consisted mainly of simulating the intervention, including “standardizing” the postoperative care. For example, in one study, a sham procedure was performed in the control arm to assess the efficacy of transplantation of embryonic dopamine neurons in Parkinson's disease. Participants receiving the sham procedure underwent a procedure of the same nature (i.e., “four twist-drill holes made through the frontal bone after local anesthesia to place tissue implants with the use of a stainless-steel guide cannula, except that the dura mater was not penetrated after the twist-drill holes were made”) [29].

For participative interventions, the sham procedures were either an attention-control intervention of the same nature or a placebo with a different mode of administration. For example, one study involved the use of hands-on procedures to simulate manipulation [30], with the participant lying prone and soft-tissue massage and gentle palpation applied to the spine, paraspinal muscles, and shoulders. In a trial assessing physiotherapy for knee osteoarthritis with an active treatment consisting of knee taping, massage, spine mobilization, and exercise, the placebo treatment was sham ultrasonography and light application of a nontherapeutic gel. Participants in the active and control arms did not attend treatment and assessment concurrently [31].

Reports of trials assessing devices also described various placebo interventions, such as use of sham prosthesis, identical apparatus, or simulation of a device. An identical apparatus consisted of an identical inactivated machine such as a detuned ultrasound machine or an activated machine with a barrier to block the treatment. For example, in a study assessing a high-strength magnetic knee sleeve for the treatment of knee osteoarthritis [32], the placebo knee sleeve was designed to provide a strong magnetic field on the surface facing away from the knee joint, thereby making it appear to offer magnetic therapy but to impart no significant magnetic field to the knee joint. Quality control procedures demonstrated that although the machine was activated, the treated area was not exposed to the treatment.

Some placebo devices were developed to mimic the actual use of a device. In a study assessing a prosorba column that involved routing the tubing that drew off the blood behind a curtain where the prosorba column was situated, in participants assigned to the sham apheresis, plasma was passed instead to a transfer bag, which had the same volume as the prosorba column. Plasma processing time was matched to that of the active treatment. Some reports described the use of opaque goggles (e.g., assessment of laser therapy), eyes covered with patches, specific positioning of participants to prevent them from seeing the treatment, or use of curtains or boxes to blind a device (see Figure 2). Because the sham procedure was not strictly identical to the experimental treatment, some authors attempted to assess the credibility of the treatment (*n* = 4).

#### Blinding to study hypothesis.

Blinding participants to the study hypothesis was reported in 12 studies (10%). This method was proposed either with the use of a sham procedure or when participants and/or health care providers could not be blinded to the treatment they received. These methods consisted of partial information given to participants (*n* = 11) and use of a modified Zelen design (*n* = 1). Partial information given to participants consisted of not informing them about the existence of a placebo, the nature of the placebo, or the purpose of the study. In a trial assessing rehabilitation for stroke participants, participants were aware that two procedures were being compared but not that one treatment was a control, because neither the consent forms nor the verbal explanations referred to the attention control intervention as a control treatment. Thus, participants could reasonably expect an improvement regardless of treatment received. One report described a modified Zelen design [33] to blind participants to the study hypothesis in a trial comparing usual care to a complex, physical therapy-based intervention for patellofemoral joint osteoarthritis of the knee [33]. First, researchers invited participants to participate in a cohort. Then they informed participants randomized to the intervention arm that they would receive the experimental treatment, and they signed a second consent form.

In one trial assessing cognitive behavioral therapy, two junior clinical psychologists, unaware of behavioral insomnia therapy, were blinded to the study hypothesis. They were not told that one of the treatments they administered was a placebo. Once all participants completed treatment, the investigative team debriefed the therapists about the study hypotheses and placebo treatment [34]. Table S2 provides examples of reporting methods of blinding participants of study hypotheses.

To avoid nonblinding because of specific treatment side effects, such as specific sensation with a transcutaneous electrical nerve stimulator), excluded participants with previous experience of the intervention, and avoided treatment crossovers, but also provided only partial information to participants of the potential manifestations linked to treatments (*n* = 6). When assessing parental auricular acupuncture [35], the research assistant informed participants that they may experience a pinching sensation with insertion of the needles and “incorrectly” informed them that this sensation was not related to group assignment.

#### Blinded centralized assessment of the primary outcome.

A total of 43 reports (35%) described specific methods to blind outcome assessors. These methods relied mainly on a centralized assessment of the main outcome. They relied on physician-driven data of a centralized assessment of clinical examination provided through video, audiotape, or photography (*n* = 9). To assess the analgesic effect of breast-feeding in term neonates compared to mothers' arms, pacifiers, placebo (i.e., sterile water), or glucose [36], the researchers videotaped the infants and the monitor screen during the procedure, and two specially trained observers independently assessed the recordings using a specific scale. The observers were blind as to the purpose and study hypothesis but had been told that they were assessing agreement of their scores in four different situations. Finally, for physician-driven data, other methods of blinding included masking a scar by a hat or sham dressing (*n* = 4) or asking participants not to tell the outcome assessor what treatment they received (*n* = 13). Table S3 describes examples of reporting methods of blinding outcome assessors.

For paraclinical examination, methods of blinding relied on a centralized assessment of complementary investigation (*n* = 13) such as centralized assessment of angiogram. For clinical events blinding relied on assessment of events by a blinded adjudication committee (*n* = 7) or a blinded assessment of the extract of the case report form by an investigator (*n* = 3).

### Classification of the Methods of Blinding

**Figure 3:**
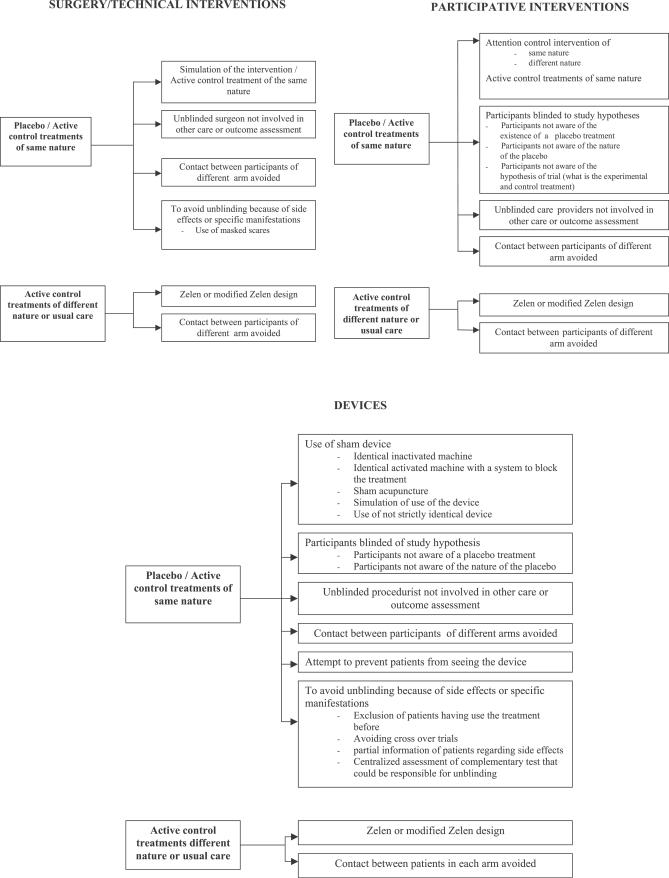
Methods of Blinding Participants, Health Care Providers, or Other Caregivers That Rely on the Category of Treatment and Comparator Assessed

**Figure 4:**
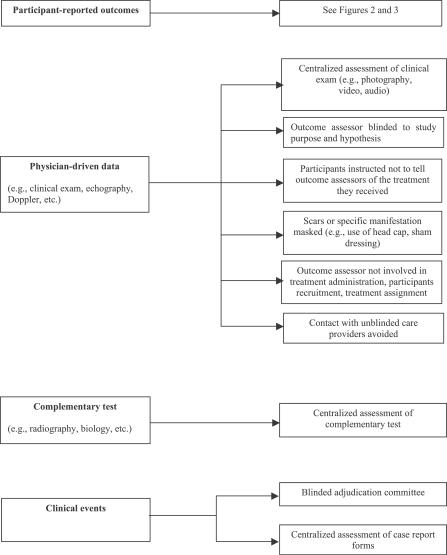
Methods of Blinding Outcome Assessors Depending on the Primary Outcome Considered

Our results helped us developed a classification scheme to distinguish between: (1) the blinding of key trial person—participants, health care providers, or other caregivers—that rely on the category of treatment (surgical/technical procedure, participative interventions, devices) and comparator assessed (Figure 3), and (2) the blinding of outcome assessors depending on the primary outcome considered (Figure 4). Although other classification systems are possible, we elected to focus on the category of treatment, the comparator, and the primary outcomes, believing them to be more practical for those planning RCTs that involve blinding. However, other ways of classifying could be analysed and discussed in further studies.

## Discussion

This study provides a detailed description and classification, with examples of reporting, of different methods of blinding participants, health care providers or other caregivers, and outcome assessors in a large series of RCTs assessing NPT. Our results highlight innovative methods of blinding and allow for a description of possible placebos for NPT in trials.

Although evidence has demonstrated that lack of blinding could bias treatment effect estimates, reports of RCTs describe blinding poorly [4,14,16,17] especially RCTs assessing NPTs [18,19]. Lack of blinding in trials assessing NPTs is probably associated, in part, with a lack of awareness of existing methods of blinding. To improve this situation, we developed an inventory of blinding methods and classified them according to what seemed reasonable after having read through the various methods. We hope that disseminating this classification scheme along with the detailed examples of reporting (Tables S1–S3) will help clinical trialists better integrate blinding into the design and conduct of their trials. As well, those critically appraising the health care literature need adequate knowledge of all possible methods of blinding to be able to determine the feasibility of blinding in a published report of an RCT [37].

Use of a placebo is a standard method to blind participants and health care providers in RCTs. Although fabrication of placebo is relatively easy for pharmacological treatment, it is more difficult for surgery, rehabilitation, or psychotherapy. Our review identified several ingenious placebo methods, such as sham surgery, sham manipulation, or sham acupuncture. However, these methods raise some issues. First, the use of sham procedures in surgery is debatable, because it is not without risk, such as with general anesthesia and intraoperative procedures. Moreover, the optimal placebo should appear exactly like the “real” treatment but lack the “supposed” specific component. Debate surrounds the use of attention-control interventions in trials assessing participative intervention, because these treatments have a specific therapeutic effect linked, for instance, to the relationship between participants and health care providers. Consequently, the use of attention-control interventions could underestimate the treatment effect [38]. Finally, use of placebo for NPT could be a barrier for the participation of patients and health care providers in such a trial. A selection bias might have been introduced in the RCT assessing arthroscopy surgery for osteoarthritis of the knee because 44% of the eligible patients declined to participate in the study. This high rate of refusal to participate probably resulted from the fact that all patients knew they had a one-in-three chance of undergoing a placebo procedure [39].

Some researchers reported using a placebo control intervention that was not identical to the active treatment, for example, in comparing an experimental rehabilitation program to sham ultrasonography. These methods could be a solution when assessing NPT. Spigt et al. [40], in assessing lifestyle advice, proposed using a syrup placebo, and assessed and validated the methodological and ethical consideration of these designs. In such trials, participants should be informed in part and blinded to the study hypothesis.

Blinding participants to study hypotheses could also be proposed when the comparator is an active control treatment of the same nature or different nature by use of a modified Zelen design [33,41]. Such design is a two-stage procedure, in which patients are asked to provide consent for an observational study in the first stage. Then patients are randomized to the experimental treatment and the control arm, and in the second stage are asked to provide a second consent for treatment. In these situations, ethical considerations require that participants be told that for scientific reasons, they were not informed about the specific goal of the study and that full information will be provided at the study's conclusion [40,42]. Further, such trials should be conducted carefully to avoid disclosure of the trial hypothesis and, especially, to avoid contact between participants in each arm. Using such a design, trialists should be aware that depending on the appearance of the experimental treatment and placebo, differences in expectations could influence the outcome, particularly for outcomes sensitive to psychological factors. In these situations, the psychological strength of the treatment could be assessed by measuring the credibility of the treatment (i.e., use of validated credibility scales [43,44]), participants' expectations (i.e., asking participants to rate their expected effect of both intervention on a Likert scale), or by registering participants' preference at the beginning of the trial before randomization [40,45–51].

Our classification scheme highlights the fact that blinding outcome assessors depends mainly on the primary outcome. We focused on the outcome because of some evidence that the risk of bias varies according to the outcome. Wood et al. [52] showed that lack of blinding yielded exaggerated treatment effect estimates for subjective outcomes but had no effect on objective outcomes. In most situations blinding outcome assessors should therefore be possible with a centralized assessment of complementary investigation, physician-mediated data, and clinical events (Figure 4). These methods are likely to be more successful than those consisting of instructing participants not to tell outcome assessors the treatment they received. In fact, a study comparing splinting and surgery in the treatment of carpal tunnel syndrome involved an attempt to blind the outcome assessor by encouraging the participants not to reveal their treatment and by masking the surgical scar. Despite these efforts, at the end of the trial assessors guessed correctly the treatment performed [53]. However, asking assessors to guess treatment assignment during a RCT may not be adequate for assessing the success of blinding [15,16,54,55]. Authors have suggested that the success of blinding should be systematically assessed in RCTs [14]. However, this recommendation was vigorously debated [15,54,56]. In fact, when asking participants to guess treatment assignment, those feeling better will tend to guess they are receiving the experimental treatment rather then the control treatment. Consequently, if the treatment is effective, the success of blinding may be discussed although the method of blinding was adequate. Blinding is more likely to be considered successful when a treatment is ineffective. Finally, for participant-reported outcomes, for which the participant is the outcome assessor, if no methods can overcome the difficulties in establishing and maintaining blinding of participants, no methods should be used so as to avoid ascertainment bias by a blinded outcome assessment.

The description of all these methods also highlights some issues such as the possible economic burden of blinding within these trials that might well be substantial and requires additional investigation. Further, some methods such as blinding patients to the study hypothesis could lead to partial blinding, and the success of these methods could be questionable [18].

### Limitations

Our study might not have captured some specific methods of blinding. In fact, we assessed only RCTs published in high-impact-factor journals during 2004. However, our aim was not to be exhaustive but to provide a description of several possible methods of blinding. Furthermore, we added reports found by searching reference lists of relevant selected articles or reports known by members of our team or experts in this field. In addition, this study assessed only the reporting of trials, not the trials themselves, and some methods might not have been reported [10,57]. However, we allowed for highlighting creative methods of blinding. Finally, the results are evolving, and readers are invited to inform us of other methods of blinding not captured in this survey.

In conclusion, we provide a classification of methods of blinding participants, health care providers, and outcome assessors in trials of NPT and a detailed description of methods that could overcome barriers to blinding in such trials. This classification should be helpful for trialists to determine the method of blinding according to the aim of the trial and for readers to assess the feasibility of blinding in and quality of RCTs assessing NPTs.

## Supporting Information

Text S1 Journals Included in the Search Strategy in PubMed (42 KB DOC)

Table S1 Description of Sham Procedures According to the Category of Treatment Assessed (154 KB DOC)

Table S2 Methods of Blinding Patients to Study Hypothesis (65 KB DOC)

Table S3 Methods to Blind Outcome Assessors (66 KB DOC)

## Abbreviations

NPT: nonpharmacological treatment
RCT: randomized controlled trial
