# Supplementary material for: Reporting Methods of Blinding in Randomized Trials Assessing Nonpharmacological Treatments
Source: PLoS Med. 2007 Feb 20;4(2):e61. doi: 10.1371/journal.pmed.0040061 (PMC1800311; doi:10.1371/journal.pmed.0040061)
Supplement: Supplementary file 1 [file pmed.0040061.sd001.doc]

Text S1 : journals included in the search strategy in PubMed

J allergy clin immun OR clin exp allergy OR "Allergy" OR pain OR "Anesthesiology" OR Br J Anaesth OR J am coll cardiol OR eur heart j OR am heart j OR brain OR ann neurol OR neurology OR Am J Respir Crit Care Med OR “Crit Care Med” OR Intensive care med OR j dent res OR Clin oral implants res OR oral oncol OR j invest dermatol OR arch dermatol OR j am acad dermatol OR ann emerg med OR acad emerg med OR am j emerg med OR diabetes OR diabetes care OR j bone miner res OR gastroenterology OR hepatology OR gut OR Am j geriatr psychiatry OR j am geriatr soc OR Drugs Aging OR circulation OR blood OR Leukemia OR antivir ther OR aids OR clin infect dis OR complement ther med OR altern ther health med OR "J Altern Complement Med" OR New England Journal of Medicine OR JAMA OR “Lancet” OR Annals of Internal Medicine OR BMJ OR Archives of Internal Medicine OR CMAJ OR Medicine OR American Journal of Medicine OR ann med OR nurs res OR cancer nurs OR Res Nurs Health OR am j clin nutr OR obes res OR j nutr OR Fertil Steril OR menopause OR hum reprod OR J. Natl. Cancer Inst OR j clin oncol OR Cancer res OR Invest Ophthalmol Vis Sci OR ophthalmology OR Arch Ophthalmol OR Osteoarthritis and Cartilage OR Spine OR J Bone Joint Surg Am OR Head Neck OR laryngoscope OR arch otolaryngol OR pediatrics OR j pediatr OR pediatr res OR hypertension OR stroke OR "Thromb Haemost" OR Arch. Gen. Psychiatry OR am j psychiat OR Biol psychiatry OR psychother psychosom OR psychosom med OR psychol med OR phys ther OR support care cancer OR arch phys med rehabil OR thorax OR J Thorac Cardiovasc Surg OR chest OR Arthritis and Rheumatism OR Ann Rheum dis OR rheumatology OR ann surg OR br j surg OR ann surg oncol OR am j transplant OR Liver Transpl OR transplantation OR trop med int health OR Trans r soc trop med hyg OR am j trop med hyg OR j am soc nephrol OR kidney int OR Am j kidney dis
