# Supplementary material for: Reporting Methods of Blinding in Randomized Trials Assessing Nonpharmacological Treatments
Source: PLoS Med. 2007 Feb 20;4(2):e61. doi: 10.1371/journal.pmed.0040061 (PMC1800311; doi:10.1371/journal.pmed.0040061)
Supplement: Supplementary file 2 [file pmed.0040061.st001.doc]

Table S1: Description of sham procedures according to the category of treatment assessed

| **Category of treatment assessed** | **Sham Procedures** |
| --- | --- |
| **Devices** | |
| High strength magnetic knee sleeve for treatment of knee osteoarthritis[1] | The experimental device was designed to saturate the entire knee joint space with a magnetic field having both high intensity and high gradients, two factors most likely to be responsible for any treatment effect of static therapy.  The placebo knee sleeve was designed to provide a strong magnetic field on the surface facing away from the knee joint thereby making it appear to offer magnetic therapy, but to impart no significant magnetic field to the knee joint.  The customized knee sleeves were made from a commercially available light-wait cotton/latex blend knee sleeve as the starting material. Each knee sleeves received two identical inserts. Each high-strength magnet insert incorporated four 1” x 0.1” nickel-plated Neodynium disc magnets and 64 partially demagnetized 0.12” x 0.06” nickel-plated Neodynium disc magnets.  Each placebo magnet insert incorporated four 1” x 0.1” brass discs and sixty four 1.12” x 0.06” nickel-plated Neodynium disc magnets.  All active and placebo inserts were constructed at our center in a standardized fashion using the appropriate metals, light-weight wood, cheesecloth, superglue, and latex encasement. An independent professional seamstress performed permanent placement of the inserts into stretch lycra pockets located on the medial and lateral aspects of the knee sleeves.  With the placebo magnet knee sleeve on, 0.001% of the knee was exposed to a maximum magnetic field intensity of 0.65G at the skin surface. However, knee tissue exposed to magnetic field of this intensity were quite limited in scope (0.03% of the knee space exposed to field greater than earth strength (0.5G))  Both high-strength and placebo magnetic sleeves emitted comparable magnetic field on their external surface upon testing with metal objects. For example, 1.9 pound of force were required to detach both the high-strength magnet sleeve and the placebo magnet knee sleeve away from a vertical refrigerator surface.  Importantly the placebo knee sleeve could not be comfortably worn inside out-therefore conversion of the placebo knee to an active knee sleeve could not occur. |
| Repetitive Transcranial Magnetic Stimulation of the right dorsolateral prefrontal cortex in post traumatic stress disorder[2]  similar example[3] | The experimental group was treated with a Magstim having a circular coil with a 9-cm diameter. The motor threshold was determined in each subject once, before treatment. This was defined as the lowest stimulation intensity capable of inducing a visible movement at least five times out of 10 stimulations.  The position of the right dorsolateral prefrontal cortex was defined as 5cm anterior (in parasagittal line) to the motor cortex. The stimulus intensity was 80% of the patient’s motor threshold intensity. Treatments were given for 20 minutes per day over 10 working days. The patients in the sham treatment group were treated in the same way as the experimental group, but the coil was held at 90° vertical over the stimulated area ( no significant magnetic field was evoked, just the auditory artifact.  No patients experience repetitive Transcranial Magnetic Stimulation before the trial. |
| Repetitive transcranial magnetic stimulation as treatment of poststroke depression[4] | An ideal sham procedure should produce the same type of acoustic artifact and scalp stimulation without producing a physiologic affect on the cerebral cortex. This should ensure that patients receiving active or sham stimulation have the same expectations regarding the efficacy of the treatment or the possibility of experiencing side effects. We have selected the technique that produces the least amount of cortical stimulation while simulating the acoustic and somatosensory elements of the stimulus. Sham stimulation was performed using similar stimulation parameters but with coil angled off the head (one-wing 90°) to produce 67%-73% reduction in the intensity of the magnetic field. This type of stimulation is not associated with activation of cortical neurons. |
| Pulsed electromagnetic fields for osteoarthritis[5] | The active apparatus with a set of light were covered and not visible to the patients. The device does not generate any noise, thermal effect, or other sensation while the treatment is administered thus allowing blinding procedure. |
| high frequency pulsed electromagnetic energy in tinnitus treatments[6] | Patients in the placebo group were treated with an inactivated machine. Blinding could be successfully performed because this device emits a non thermal, pulsed high frequency electromagnetic energy that could not be felt. Both the active and placebo treatments were identical in appearance, sounds, and presence of a light when activated. |
| Magnetic insoles on heel pain[7] | The sham-magnetic insoles were identical to the active insoles, but were specially made by the manufacturer with the same metal foil, but in a nonmagnetized state. All insoles were provided by the manufacturer in a box and were completely indistinguishable by appearance, touch. Participants were required to sign an agreement to refrain from covert attempts to determine whether the insoles they were given had active magnetic properties. |
| Percutaneous electrical nerve stimulation[8] | Patients were told that we were comparing 4 different NPT for LBP. The sham treatment consisted of the placement of 10 acupuncturelike needle probes in an identical montage then the active treatment, but no electrical stimulation was applied to the probes. |
| Effect of electric stimulation on C and A delta fiber-mediated thermal perception[9] | The control group underwent precisely the same preparation as subjects in the experimental groups with the electrodes being applied in the same manner. |
| Transcutaneous Electrical nerve Stimulation (TENS) for chronic pain[10] | Both real and sham unit were provided by the manufacturer and had “on” lights that flashed at the stimulation frequency set by the control knobs, and a battery indicator light that flashed when batteries were low. All patients in both groups received the same content of batteries and rechargers, same unit and same instructions.  To enhance blinding, any prior use of TENS was an exclusion criteria, cross over design were avoided, the clinicians performing follow-up examinations, the therapist responsible for instruction patients and applying TENS specifically asked subjects not to discuss their therapy with examiner. The examining clinician who performed outcome assessment was different from the examining clinician at baseline. Follow-up schedule, and follow-up visit duration were similar for the 2 groups as well as all written and verbal instructions. |
| Pulse electric stimulation[11] | Use of identical appearing placebo device. The device initially produce an electrical impulse at startup the same as the active device. The voltage of the placebo was then adjusted to the subthreshold level using the same procedure as the active device until the tingling sensation was no longer felt. After the subthreshold level was set, the placebo device output switched off. |
| TENS and osteoarthritis[12] | Cross over RCT. Adaptors were plugged into the ends of electrode leads either to allow the current to flow to the patient’s knee or to block its flow (placebo).Adjustment of TENS intensity aimed at obtaining the highest amplitude with continuing comfort. Patients were not told what the TENS for each treatment phase would feel like. Patients were reassured that whatever they were feeling was normal and appropriate. |
| Deep brain stimulation[13] | A permanent electrode containing four contact sites was implanted with the patient under local anesthesia and was connected to a pulse generator that was placed subcutaneously in the subclavicular area with the patient under general anesthesia. The pulse generator could be programmed and the investigator and the patients were unaware of whether the stimulation had been on or off. |
| Deep brain stimulation[3] | Same as above |
| Effect of multiwavelength light therapy in the treatment of pressure ulcers[14] | Treatment group received 14 sessions of multiwavelength light therapy. The laser device was calibrated before each treatment session. During treatment, the eyes of all subjects and of the therapist were covered with protective goggles. These precautions were carried out for both treatment and control groups. The treatment group received multiwavelength light therapy, whereas for the control group the multiwave light therapy source was held over the ulcer after switching off the beam.  Pressure ulcers in both groups received daily dressing with sterile gauze soaked in normal saline and pressure relief with either a water mattress or a split mattress. The dressing were given by the neurologic rehabilitation ward nursing staff, who was blinded to the treatment status. |
| Electrohydraulic high-energy shock-wave treatment for chronic plantar fasciitis[15] | A minimum of two physicians investigators participated at each study site. One investigator served as treatment-blinded evaluator and was not allowed to observe patient treatment. The actual procedure was performed by a second trained physician who was aware of the specific treatment rendered, but who did not play any role in evaluating the patient.  For patients assigned to placebo, a Styrofoam block was placed against the treatment heat to absorb the shock waves by the presence of multiple air cavities. In addition, a fluid-filled intravenous bag was placed between the Styrofoam block and the subject’s heel to mimic the waterfilled treatment head. Patients who received a placebo treatment did not have any coupling gel applied to the heel. Placebo patients also had 1500 shocks “delivered” according to the aformentioned protocol, effectively duplicating the duration and noise of an active treatment. Patients who received active and placebo treatments were kept apart in the recovery room to avoid any discussions and comparisons about what occurred in the surgical suite. |
| Extracorporeal Shock Waves Therapy for the treatment of Chronic Calcifying Tendonitis of the Rotator Cuff.[16] | A shock wave head was coupled to the shoulder with a thin sheet of polyethylene foil placed between the shock wave head and the patient. Coupling gel was used between the shock wave head and the foil and between the foil and the shoulder. In the sham treatment an air chambered polyethylene foil with coupling gel was placed against the patient skin, but no coupling gel was applied to the site of the shock wave head. In every other respect the setup was the same, with same frequency intensity of shock waves. Measurements with glassfiber hydrophones demonstrates that no shocks waves could pass through the foil. The patients’ prone position prevented them from seeing the device, but they could hear the typical sound of shock waves being generated. |
| Treatment of diabetic peripheral neuropathy with the Anodyne Therapy System, a near-infrared medical device[17] | Treatments were administered with the ATS Model 480, supplied for this study by its manufacturer. The ATS is a medical device consisting of a base power unit and therapy pads containing 60 near-infrared gallium aluminium arsinide diodes used to increase circulation by dilating arteries and veins. Inactivating the diodes so that no near-infrared photo energy was emitted and inserting heaters preset at 37°C created sham devices of identical appearance. Only the manufacturer knew the treatments allocated |
| Low intensity laser therapy for painful symptoms of diabetic sensorimotor polyneuropathy[18] | Two of four instruments were modified by the manufacturer to prevent generation of laser energy. Externally, the instruments were indistinguishable. The output of the laser instruments was measured and recorded by the project administrator on a weekly basis. |
| Pulsed-dye laser and acne vulgaris[19] | Controls were treated with disconnected laser handpiece that was moved across the face in an identical manner to that for the intervention group. All patients wore opaque googles during treatment to protect their eyes and to ensure that they were unaware of the therapy they received. Treatment was given in locked room with no windows.. |
| Radiotherapy for exsudative age-related macular degeneration[20] | Treatment: conventional simulation followed by computed tomography (CT) localization or CT simulation. 7 fractions of 2 Gy administred during 7 working days.  The sham group did not undergo CT simulation and received only 1 sham session. Sham session was identical than active session. Both groups adhered to identical follow-up schedules. |
| Radiotherapy for Graves’ orbitopathy[21] | intervention 20 Gy in 10 fractions  sham intervention 0 Gy in 10 fractions. Exactly the same procedure (positioning, of the patients and of the device, simulator). Sham irradiation was carried out with the 4 MeV electrons, as the collimator blocks were closed and a cerrosafe block was placed on a tray in front of the residual gap to prevent leakage of irradiation. Measurements proved the dose to be undetectable apart from the background level. The apparatus was started up during sham irradiation so that patients would not know which treatment they received. The type of treatment administered was know only in the department of radiotherapy. |
| Pacemaker implantation for the risk of syncope relapse[22] | Patients were randomized to pacemakers ON with active (DDD) mode with rate-drop response (RDR) or pacemaker OFF in the inactive (OOO) mode. In each hospital there was only one person responsible for programming the pacemaker. |
| Pace maker[23] | An unblinded nurse or physician who had no other patient contact did all the programming. Blinded study personnel were asked not to perform routine electrocardiogram. |
| Acupuncture for disabling breathlessness[24] | Patients were randomized to one of the following two treatment sequences; real acupuncture followed by mock TENS; or mock TENS followed by real acupuncture. Blinding was not possible for the therapist but treatment credibility, both in this study and the previous pilot, was evaluated as a surrogate for patient blinding. The use of Mock TENS as a placebo/control has been applied successfully, effectively, and credibly in previous studies[25].  Treatment credibility was evaluated using a well validated credibility scale[26]. |
| Parental auricular acupuncture to reduce parental preoperative anxiety and thus allow children to benefit from parental presence during induction of anaesthesia[27] | Acupuncture group: Mother in this group received 3 occlusive auricular press needles at the external ear ipsilateral to the dominant hand. All these ear points have been documented previously to reduce state of anxiety.  Sham control group: : Mother in this group received 3 auricular press needles at the external ear ipsilateral to the dominant hand. None of these 3 ear points is known to reduce anxiety. |
| Acupuncture for knee osteoarthritis[28] | The standard acupuncture intervention entailed the insertion of sterile, single use, 30 gauge and 45 mm length acupuncture needles into the local points. For each of the points in question, the acupuncturist determined the patient’s sensation of Deqi (an elicitation of needle sensation to check that the puncture was performed in the correct site). A WQ-10D1 electrostimulator was used to stimulate all the needles inserted into the local points electrically, in pairs.  The same specialists carried out the placebo acupuncture, at the same frequency, and for the same duration as for the group receiving the true intervention. Retractable needles went into small adhesive cylinders, such that needles was supported but did not perforate the skin[29]. The acupuncturist then placed the needles over the same points as were used for the true acupuncture group. He connected the same pairs of electrodes and simulated the electrical connection. |
| Acupuncture for seasonal allergic rhinitis[30] | In the control group patients were treated using standardized non-acupuncture points chosen because they were distant from any “meridians”. Needling in this group was superficial and exclusively intracutaneous and needling sensation (“De-Qi”) was avoided. Only small needles were selected and physicians were instructed to avoid any form of manipulation. Therapeutic setting, procedure, and frequency of sessions were similar in both groups. |
| Acupuncture for neck pain[31] | A well validated placebo that could not have a specific physiologic effect was used[29].Electrodes were fixed to the patients’ skin and connected to the stimulator through decommissioned cables. The cables were severed inside the plug, so that no current could reach the patients. The electroacupuncture stimulator emits visual and audio signal. Patients were told that the machine could stimulate acupuncture points through high frequency, low intensity stimulation and therefore would not produce any sensation. If patients reported sensation, the therapist adjusted the unit for comfort although since this was a sham procedure, such adjustments made no real difference. |
| Acupuncture for allergic rhinitis[32] | Children with prior acupuncture experience were excluded, to ensure that those who received sham acupuncture did not notice the absence of sensation of Qi during the sham acupuncture. For sham acupuncture, the same acupoints were punctured with the same acupuncture needles; however, the needles were not inserted to a depth of only 0.3cm and no manipulation of the needle was performed to avoid qi. Same number of session. |
| Acupuncture after oral surgery[33] | In the placebo group, the procedure was identical to that used in the intervention group, but without needle insertion into the skin[29]. Manipulations were made by palpating the surface of the skin with a blunt dental instrument at the same 3 points in time as the treatment group. In both groups the patients’ eyes were covered with patches so they could not view the treatment procedure. A pair of electrodes from a mock electrical stimulator was attached to the end of the needles in the real and placebo acupuncture group. |
| Electrical vs manual acupuncture[34] | For patients in the manual acupuncture, devices were applied without electrical stimulation. The device was programmed by an independent unblinded technician. |
| Acupuncture for pain[35] | intervention group: electroacupunture  sham group: the device was switched on, but no needles were inserted into the patients. Acupuncture was done by an experienced acupuncturist who was not involved in data acquisition, analysis or other treatment delivery. The site of acupuncture was covered from the research team by curtains. |
| Electro acupuncture for knee osteoarthritis[36] | The placebo EA was performed by attaching patch electrodes to the selected acupuncture points. Each electrode was connected to the sound producing dummy mode of the same apparatus, as in the true EA treatment. The duration and frequency of treatment were similar to the active EA group. Only the acupuncturist was unblinded. |
| Acupuncture for pain[37] | use of same acupoint, use of specific needles[29]. Patients informed that 2 types of needles were tested: a conventional needle and a new needle, either of which may be successful in treating their pain. |
| Knee tape for osteoarthritis[38] | 3 arms: active tape, control tape, no tape. Patients were unaware of which taping technique was considered therapeutic. Control tape aimed at providing sensory input only. Hypoallergenic tape alone was laid over the same area of skin as the therapeutic tape but did not use underloading of infrapatellar fat pad and medial patellar glide and tilt, anteroposterior tilt. |
| Laterally elevated wedged insoles in the treatment of knee osteoarthritis[39] | The control treatment is a neutrally wedged insole.  Patients were informed that the study aimed to compare two kinds of insoles, but not that one was presumed to be of greater efficacy than the other. This is not a full informed consent, but this approach has been considered by the investigators as ethical since it has been suggested that neutrally wedged insoles might relieve some symptoms by absorbing impact load. |
| Heat retaining knee sleeves on joint pain[40] | Placebo sleeves were of similar cotton/elastane construction but did not contain the aluminium-coated substrate and fiber matrix. The placebo sleeve was comparable in weight, and provided a similar area of contact to control for proprioceptive effects. Sleeves were dispensed to patients in plain unmarked boxes. |
| Active warming during emergency transport and low back pain[41] | Patients were all covered with a carbon-fiber electric heating blanket which was in turn covered by a single woolen blanket. Resistive heating was induced by running a 7 to 8 ampere current through the carbon fiber, batteries providing sufficient power for up to 40 minutes of heating. According to the randomization, the heating system was set by an unblinded investigator who subsequently locked the control unit in a metal box. For the patients assigned to active warming, the blanket temperature was set to 42°C. In contrast, the heating system was not activated for patients assigned to passive warming. |
| Drinking water device[42] | Use of externally identical devices that differ only in their ability to remove microorganisms from water. |
| Impermeable covers and allergic rhinitis[43] | Each patient took his box containing the active or inactive cover and home and opened it in the absence of the research staff |
| Prosorba column for rheumatoid arthritis[44] | An unblinded operator was responsible of the treatment and the tubing that drew off their blood was routed behind a curtain, where the prosorba column was situated. The unblinded operator turned stop cocks to the appropriate position so that plasma from the patient flowed either through the Prosorba column or in a bypass loop around the column. For patients assigned to the sham apheresis, plasma was passed, instead, to a transfer bag, which had the same volume as the Prosorba column. Plasma processing time was matched to that of the active treatment. In both treatment arms, processed plasma was returned to the patient through a leucocyte removal filter capable of removing microaggregates. |
| Bispectral index monitoring to prevent awareness during anaesthesia compared to usual care [45] | A BIS sensor was applied to every patient’s forehead before induction of anaesthesia and connected to a monitor that was concealed from patients. In patients allocated to usual care, the monitor was turned off. In patients allocated to BIS; the delivery of anaesthesia was adjusted to maintain BIS of 46-60 from the start of laryngoscopy to the time of wound closure. |
| Heat wrap applied to the wrist for wrist pain[46] | Heat wrap (40°C) within 30 minutes of exposure to air and maintains this temperature continuously for an 8-hour period of wear. The placebo group received unheated wrap. |
| Intracoronary gamma radiation therapy for In-Stent Restenosis[47] | A closed en-lumen radiation delivery catheter was inserted into the vessel and positioned to span the lesion length. The patient was randomly assigned to receive a nylon ribbon containing different seed trains of either placebo or 192-Ir.The radiation oncologist hand-loaded the ribbon from a lead container positioned on a cart next to the table into the closed end-lumen catheter. The cardiologist documented accurate positioning of the source by angiography. All catheterization laboratory personnel left the room during the dwell period for active source radiation or placebo treatment except for the radiation safety officer, who measured exposure rates at various locations. At the end of the treatment, the radiation oncologist entered the room and retrieved the ribbon into the shielded lead container and the medical personnel returned once the radiation exposure reached background values. |
| **SURGERY and technical interventions** | |
| Tidal irrigation for knee osteoarthritis[48] | A vertical drape was shield to mask the patients’ view and the nurse assessor was excluded from the room until the procedure and clean up were completed. The contact between patients and the investigator / procedurist was limited to the day of the procedure.  Same setting, cleansing, topical anesthesia. A 1 liter bag of steril normal saline was hung at the edge of the drap within view of the patients. The knee was then draped with steril towels and the connecting tubing were assembled and attached to an empty 1-liter waste bag and a 50-ml syringes, producing a closed system for fluid delivery, aspiration and ejection. To administer the sham treatment, a 14-gauge needle was advanced to, but not through, the joint capsule via the lateral port. Fresh saline was drawn from the supply bag in aliquots of 40-50ml and 3-5ml of saline was clysed into the subcuraneus tissue with ceah mimicking “exchange” before the remainder of the saline was expelled into the waste bag. Positioning of the knee and manipulation were performed as for active treatment. After passage of 1 liter of saline through the tubing, the needle was removed. |
| Transplantation of embryonic dopamine neurons[49] | Only the surgical team was aware of treatment assignment. After a stereotactic ring was fixed to the skull, magnetic resonance imaging was used to established coordinates for 4 needles passes in the axial plane of the putamen. Surgery was performed with patients awake, with local anesthesia administered to the skin of the forehead.4 twist-drill holes through the frontal bone were made along the planned axis of the tracks. The tissue implants were placed with the use of a stainless-steel guide cannula with a graduated outer diameter of 1.5-0.6mm.  The patients in the sham procedure underwent an identical procedure except that the dura mater was not penetrated after the twist-drill holes had been made in the frontal bone. |
| Radiofrequency lesioning of dorsal root ganglia for lumbosacral radicular pain[50] | Same intervention performed by anaesthesists but the radiofrequency generator technician was instructed whether or not to apply current. The technician operated the radiofrequency generator with the display turned away from the operating table so that no visual or auditory signals could be conveyed to the patient or treating doctor. |
| Arthroscopy surgery for knee osteoarthritis[51] | Patients in the interventions arms received general anaesthesia with endotracheal intubation. Patients in the placebo group received a short acting intravenous tranquilizer and an opioid and spontaneously breathed oxygen-enriched air. To preserve blinding in case of partial anesthesia, a standard arthroscopic debridement procedure was simulated: the knee was prepped, draped and 3 1-cm incisions were made in the skin. The surgeon asked for all instruments and manipulated the knee as if arthroscopy were being performed.Saline was splashed to simulate the sound of the lavage. No instrument entered the portals for arthroscopy. The patients were kept in the operating room for the amount of time for a debridement. patients spent the night after the procedure in the hospital and were cared for by blinded nurses. |
| Effect of endoscopic therapy for bleeding ulcers[52] | Sham endoscopic treatment consisted of gentle irrigation of the ulcer base using a syringe but no manipulation with the heater probe, minisnare or suction. The endoscopists were not involved in subsequent patients managements |
| Laparoscopic adhesiolysis for chronic abdominal pain[53] | All patients received diagnostic laparoscopy, the intervention group received also adhesiolysis and the control group received no treatment. |
| Arthrographic joint distension[54] | One radiologist performed all interventions according to a standardized protocol. Care was taken to ensure that all participants in both groups were handled in an identical manner: same position for patients, same position of the image intensifier, same mark on the skin for arthrogram needle site and same local anaesthetic. The arthrogram needle was positioned, connected to connector tap and tube, and 0.5-1 ml of contrast injected to confirm the needle position. A further 6 ml of contrast was injected and an X ray picture taken, but no injection of depomedrol of distention with saline injection. Same rehabilitation program after the procedure. |
| Autologous transplanted epidermal cell suspensions for repigmenting vitiligo[55] | Same intervention but use of either autologous transplanted epidermal cell suspensions (melanocytes medium+hyaluronic acid+epidermal cell) or placebo suspension (melanocytes medium+hyaluronic acid) |
| **Rehabilitation / diet / psychotherapy / education** | |
| Efficacy of Eplet maneuver for vertigo[56] | Patients were told during the consent process that if they received the placebo and did not improve, they would receive the active treatment at the end of the trial.  Use of a placebo maneuver: the placebo patients were asked to sit and then to lie down for 5 minutes and then to return in the initial position. |
| Manipulation[57] | Active chiropractic treatment consisted of manipulation (adjustments) with the subject prone, lying on one side, and supine, in conjunction with the administration of gentle soft-tissue therapy to the overlying tissues. The specifics of treatment for each subject (vertebral segments treated, direction and type of manipulation, and use of soft-tissue therapy) were determined by the treating chiropractor. All the chiropractors used the diversified technique in common use in Canada and the United States, which involves manual contact with spinal or pelvic joints followed by a low-amplitude, high-velocity directional push often associated with joint opening, creating a cavitation, or "pop."  For simulated treatment, the subject lay prone while soft-tissue massage and gentle palpation were applied to the spine, paraspinal muscles, and shoulders. A distraction maneuver was performed by turning the subject's head from one side to the other while alternately palpating the ankles and feet. The subject was positioned on one side, a nondirectional push, or impulse, was applied to the gluteal region, and the procedure was repeated with the subject positioned on the other side; then the subject was placed in the prone position, and a similar impulse was applied bilaterally to the scapulae. The subject was then placed supine, with the head rotated slightly to each side, and an impulse applied to the external occipital protuberance. Low-amplitude, low-velocity impulses were applied in all these nontherapeutic contacts, with adequate joint slack so that no joint opening or cavitation occurred. Hence, the comparison of treatments was between active spinal manipulation as routinely performed by chiropractors and hands-on procedures without adjustments or manipulation. |
| Physiotherapy for knee osteoarthritis[58] | Active treatment consisted of knee taping, massage, spine mobilization, and exercices  Placebo treatment consisted of sham ultrasound and light application of non therapeutic gel. patients in the active arm and control arm did not attend for treatment or assessment concurrently.  Physiotherapist implemented both active and placebo treatments. During the follow-up period there was no contact between physiotherapists and patients. |
| Home based training for knee ostoarthritis[59] | Intervention: Home based progressive strength training program + home visits 12 visits over 4 months + booklet, + kept a log  The attention control group served 2 purposes: to provide a placebo intervention and to simulate the contact patients in the experimental group received at home. Although nutrition was not the active intervention, patients were informed we were examining the effects of both exercise and nutrition. Nutrition education was chosen because it is associated with arthritis it was believable and this intervention suppose behavior change similar to exercise. The attention control group received a booklet based on eating and home visit every 2 weeks for a total of 7 home visits over 4 months. Topics covered an introduction and an overview of the program, fruit and vegetable, grains and fibers, calcium and fat. Goals were set and patients were asked to keep food logs that were reviewed at each follow-up visit. |
| Food elimination for irritable bowel syndrome[60] | Staff Laboratories produced a true and sham diet sheet for each patient. The sham diet eliminated the same number of foods to which a patient exhibited IgG antibodies but not those particular foods. The goal was to try and include in the sham diet an equally difficult to eliminate staple food for every staple food in the true diet. Thus cow milk was replaced with potato, wheat with rice, and yeast with whole egg.Nut activities were replaced with other nuts in the sham diet and legumes with ather legumes. |
| Oats for celiac disease[61] | To ensure blinding, oats were mixed with otherwise gluten-free products such as bread…. |
| Korean hand acupressure[62] | The sham group received acupressure at a sham point defined as an acupuncture point known to have no effect. |
| Reflexology[63] | Simulated reflexology was given in a similar way using “placebo” area on the feet, extremities and backs, the active area being avoided. |
| Cognitive behavioral therapy[64] | Enrollees were blinded to hypotheses and the nature of the placebo, but they were told they had 1 in 3 chance of placebo assignment. Therapist were blinded of hypothesis and were told that 1 of the treatment they administered was a placebo. Therapists were 2 beginning level clinical psychologists, naïve to behavioral insomnia therapy. Before treating study participants they were required to review the project’s treatment manual and audio recordings demonstrating treatments, and then show competence with each treatment via role play sessions with the first author. Throughout the project, therapists provided their assigned participants 6 weekly, 30- to 60-minute individual sessions of their respective treatments. The first author jointly supervised the therapists every 8 to 12 weeks throughout the study to ensure their adherence to treatment protocol and to counter any emerging suspicions about the hypotheses or placebo condition. Once all enrollees completed treatment, therapists were debriefed about study hypotheses and placebo treatment.  The patients assigned to PT received a quasi-desensitization treatment,*^[52](http://jama.ama-assn.org/cgi/content/full/285/14/#REF-JOC01726-52)^* presented as a means of eliminating the "conditioned arousal," which prolongs nocturnal awakenings. Therapists helped each PT recipient develop a chronological 12-item hierarchy of common activities he/she did on awakening at night (eg, opening eyes, clock watching). Therapists also helped them develop 6 imaginal scenes of themselves engaged in neutral activities (eg, reading the newspaper). Each session, PT recipients were taught to pair neutral scenes with items on the 12-item hierarchy so, by the end of the sixth session, all hierarchy items had been practiced with therapist assistance. Each session, the exercise was tape recorded and the patient was given this tape locked in a player like the device provided to the RT recipients. The patients assigned to PT were told to practice their exercises at home once each day, no less than 2 hours before bedtime, but to avoid using the tape or exercise during sleep periods. |
| Education to improve medication adherence[65] | The control group had shorter educational session, with a non-interactive, text only computer program that provided basic information about the disease, but not its management, possible drug side effects, or anything about patient involvement in decision regarding taking the medication. |
| Manual therapy for back and neck complaints[66] | Placebo treatment consisted of a physical examination and subsequently short-wave diathermy (10 minutes) and detuned ultrasound carried out by a physiotherapist. |
| Home based exercise[67] | Sham exercise program consisted of non-weight bearing joint unloading and stretches that did not include resistance or progression. Exercise session, injuries, and other health problems preventing participation in exercise were recorded in diary. |
| **Other treatments** | |
| Effect of Qi therapy on premenstrual syndrome[68] | Subjects were informed that they would receive one of 2 types of qi, each of which had the potential to relieve premenstrual syndrome.  qi is a concius intent to help the patients while becoming mentally aware of the self as one with the cosmos.  Sham treatment was administered by the same therapist who aimed to mimic the gestures used in the actual qi therapy without any effort or intention to emit real qi. She followed the experiment schedules and attempted to emit qi with positive thinking to restore harmony and balance to the energy systems of the subjects or mimicked it using the same protocol so that subjects were unaware of her intentions. She was not involved in other aspects of the study. |
| effect of hemispheric synchronization on intraoperative analgesia[69] (n°81) | After endotracheal intubation , positioning and adequate hypnosis, headphones were placed on patients’ ears and the tape was turned on. Patients were assigned Hemi-Sync or blank tape. |
| Preoperative autologous blood donation (PABD) compared to acute normovolemic hemodilution (ANH) [70] | After the patient was in the supine position, the brachial vein in one arm was exposed for venipuncture. The site was shielded from the patient’s vision by use of a suspended drape. In both groups, a large-bore IV catheter was inserted in the brachial vein. Blood was collected or no collection occurred in the placebo group. In both groups, patients were assessed for vital signs and were given ferrous sulfate.  Each bag still had a connected satellite pack, and it was used to remove 100-150 ml of plasma, such that the bag volume was the same as that of standard allogeneic unit. On the morning of surgery, 3 unit of blood (autologous in the PABD group, and allogenic in the ANH group) were identified for each patient. For each bag the upper left hand front corner of the blood bag indication that the bag contains autologous blood was obscured with a prepared sticker which could be removed at a later date. A special screen hiding all components of the unit was used in all case so that anaesthesists and surgeons could see only the perfusionists and the distal lines of the circuit. The oxygenator, the reservoir and its filling system were not visible to physicians. |
| Hyperbaric oxygen therapy[71] | Not detailed |
| Blood patch[72] | An unblinded anesthesiologist investigator injected the autologous blood through the epidural catheter or pretended to inject the blood but did not actually made an injection over a several minute period. At the end of the injection period, the catheter and syringes were dropped onto an opaque towel behind the back of the subjects. The towel was wrapped around the catheter and syringe and disposed of so that the subject and her family members, physicians, and nurses were blinded. |

**References**

1. Wolsko PM, Eisenberg DM, Simon LS, Davis RB, Walleczek J, et al. (2004) Double-blind placebo-controlled trial of static magnets for the treatment of osteoarthritis of the knee: results of a pilot study. Altern Ther Health Med 10: 36-43.

2. Cohen H, Kaplan Z, Kotler M, Kouperman I, Moisa R, et al. (2004) Repetitive transcranial magnetic stimulation of the right dorsolateral prefrontal cortex in posttraumatic stress disorder: a double-blind, placebo-controlled study. Am J Psychiatry 161: 515-524.

3. Loo C, Mitchell P, Sachdev P, McDarmont B, Parker G, et al. (1999) Double-blind controlled investigation of transcranial magnetic stimulation for the treatment of resistant major depression. Am J Psychiatry 156: 946-948.

4. Jorge RE, Robinson RG, Tateno A, Narushima K, Acion L, et al. (2004) Repetitive transcranial magnetic stimulation as treatment of poststroke depression: a preliminary study. Biol Psychiatry 55: 398-405.

5. Trock DH, Bollet AJ, Markoll R (1994) The effect of pulsed electromagnetic fields in the treatment of osteoarthritis of the knee and cervical spine. Report of randomized, double blind, placebo controlled trials. J Rheumatol 21: 1903-1911.

6. Ghossaini SN, Spitzer JB, Mackins CC, Zschommler A, Diamond BE, et al. (2004) High-frequency pulsed electromagnetic energy in tinnitus treatment. Laryngoscope 114: 495-500.

7. Winemiller MH, Billow RG, Laskowski ER, Harmsen WS (2003) Effect of magnetic vs sham-magnetic insoles on plantar heel pain: a randomized controlled trial. Jama 290: 1474-1478.

8. Ghoname EA, Craig WF, White PF, Ahmed HE, Hamza MA, et al. (1999) Percutaneous electrical nerve stimulation for low back pain: a randomized crossover study. Jama 281: 818-823.

9. Palmer ST, Martin DJ, Steedman WM, Ravey J (2004) Effects of electric stimulation on C and A delta fiber-mediated thermal perception thresholds. Arch Phys Med Rehabil 85: 119-128.

10. Deyo RA, Walsh NE, Schoenfeld LS, Ramamurthy S (1990) Can trials of physical treatments be blinded? The example of transcutaneous electrical nerve stimulation for chronic pain. Am J Phys Med Rehabil 69: 6-10.

11. Zizic TM, Hoffman KC, Holt PA, Hungerford DS, O'Dell JR, et al. (1995) The treatment of osteoarthritis of the knee with pulsed electrical stimulation. J Rheumatol 22: 1757-1761.

12. Lewis B, Lewis D, Cumming G (1994) The comparative analgesic efficacy of transcutaneous electrical nerve stimulation and a non-steroidal anti-inflammatory drug for painful osteoarthritis. Br J Rheumatol 33: 455-460.

13. (2001) Deep-brain stimulation of the subthalamic nucleus or the pars interna of the globus pallidus in Parkinson's disease. N Engl J Med 345: 956-963.

14. Taly AB, Sivaraman Nair KP, Murali T, John A (2004) Efficacy of multiwavelength light therapy in the treatment of pressure ulcers in subjects with disorders of the spinal cord: A randomized double-blind controlled trial. Arch Phys Med Rehabil 85: 1657-1661.

15. Ogden JA, Alvarez RG, Levitt RL, Johnson JE, Marlow ME (2004) Electrohydraulic high-energy shock-wave treatment for chronic plantar fasciitis. J Bone Joint Surg Am 86-A: 2216-2228.

16. Gerdesmeyer L, Wagenpfeil S, Haake M, Maier M, Loew M, et al. (2003) Extracorporeal shock wave therapy for the treatment of chronic calcifying tendonitis of the rotator cuff: a randomized controlled trial. Jama 290: 2573-2580.

17. Leonard DR, Farooqi MH, Myers S (2004) Restoration of sensation, reduced pain, and improved balance in subjects with diabetic peripheral neuropathy: a double-blind, randomized, placebo-controlled study with monochromatic near-infrared treatment. Diabetes Care 27: 168-172.

18. Zinman LH, Ngo M, Ng ET, Nwe KT, Gogov S, et al. (2004) Low-intensity laser therapy for painful symptoms of diabetic sensorimotor polyneuropathy: a controlled trial. Diabetes Care 27: 921-924.

19. Seaton ED, Charakida A, Mouser PE, Grace I, Clement RM, et al. (2003) Pulsed-dye laser treatment for inflammatory acne vulgaris: randomised controlled trial. Lancet 362: 1347-1352.

20. Marcus DM, Camp MW, Sheils WC, McIntosh SB, Leibach DB, et al. (1999) Sham radiation in clinical trials assessing radiotherapy for exudative age-related macular degeneration. Retina 19: 525-530.

21. Mourits MP, van Kempen-Harteveld ML, Garcia MB, Koppeschaar HP, Tick L, et al. (2000) Radiotherapy for Graves' orbitopathy: randomised placebo-controlled study. Lancet 355: 1505-1509.

22. Raviele A, Giada F, Menozzi C, Speca G, Orazi S, et al. (2004) A randomized, double-blind, placebo-controlled study of permanent cardiac pacing for the treatment of recurrent tilt-induced vasovagal syncope. The vasovagal syncope and pacing trial (SYNPACE). Eur Heart J 25: 1741-1748.

23. Connolly SJ, Sheldon R, Thorpe KE, Roberts RS, Ellenbogen KA, et al. (2003) Pacemaker therapy for prevention of syncope in patients with recurrent severe vasovagal syncope: Second Vasovagal Pacemaker Study (VPS II): a randomized trial. Jama 289: 2224-2229.

24. Lewith GT, Prescott P, Davis CL (2004) Can a standardized acupuncture technique palliate disabling breathlessness: a single-blind, placebo-controlled crossover study. Chest 125: 1783-1790.

25. Davis CL, Lewith GT, Broomfield J, Prescott P (2001) A pilot project to assess the methodological issues involved in evaluating acupuncture as a treatment for disabling breathlessness. J Altern Complement Med 7: 633-639.

26. Devilly GJ, Borkovec TD (2000) Psychometric properties of the credibility/expectancy questionnaire. J behav ther exp psychiatry 31: 73-86.

27. Wang SM, Maranets I, Weinberg ME, Caldwell-Andrews AA, Kain ZN (2004) Parental auricular acupuncture as an adjunct for parental presence during induction of anesthesia. Anesthesiology 100: 1399-1404.

28. Vas J, Mendez C, Perea-Milla E, Vega E, Panadero MD, et al. (2004) Acupuncture as a complementary therapy to the pharmacological treatment of osteoarthritis of the knee: randomised controlled trial. Bmj 329: 1216.

29. Streitberger K, Kleinhenz J (1998) Introducing a placebo needle into acupuncture research. Lancet 352: 364-365.

30. Brinkhaus B, Hummelsberger J, Kohnen R, Seufert J, Hempen CH, et al. (2004) Acupuncture and Chinese herbal medicine in the treatment of patients with seasonal allergic rhinitis: a randomized-controlled clinical trial. Allergy 59: 953-960.

31. White P, Lewith G, Prescott P, Conway J (2004) Acupuncture versus placebo for the treatment of chronic mechanical neck pain: a randomized, controlled trial. Ann Intern Med 141: 911-919.

32. Ng DK, Chow PY, Ming SP, Hong SH, Lau S, et al. (2004) A double-blind, randomized, placebo-controlled trial of acupuncture for the treatment of childhood persistent allergic rhinitis. Pediatrics 114: 1242-1247.

33. Lao L, Bergman S, Hamilton GR, Langenberg P, Berman B (1999) Evaluation of acupuncture for pain control after oral surgery: a placebo-controlled trial. Arch Otolaryngol Head Neck Surg 125: 567-572.

34. Sator-Katzenschlager SM, Scharbert G, Kozek-Langenecker SA, Szeles JC, Finster G, et al. (2004) The short- and long-term benefit in chronic low back pain through adjuvant electrical versus manual auricular acupuncture. Anesth Analg 98: 1359-1364, table of contents.

35. Meissner W, Weiss T, Trippe RH, Hecht H, Krapp C, et al. (2004) Acupuncture decreases somatosensory evoked potential amplitudes to noxious stimuli in anesthetized volunteers. Anesth Analg 98: 141-147, table of contents.

36. Sangdee C, Teekachunhatean S, Sananpanich K, Sugandhavesa N, Chiewchantanakit S, et al. (2002) Electroacupuncture versus diclofenac in symptomatic treatment of osteoarthritis of the knee: a randomized controlled trial. BMC Complement Altern Med 2: 3.

37. White P, Lewith G, Hopwood V, Prescott P (2003) The placebo needle, is it a valid and convincing placebo for use in acupuncture trials? A randomised, single-blind, cross-over pilot trial. Pain 106: 401-409.

38. Hinman RS, Crossley KM, McConnell J, Bennell KL (2003) Efficacy of knee tape in the management of osteoarthritis of the knee: blinded randomised controlled trial. Bmj 327: 135.

39. Pham T, Maillefert JF, Hudry C, Kieffert P, Bourgeois P, et al. (2004) Laterally elevated wedged insoles in the treatment of medial knee osteoarthritis. A two-year prospective randomized controlled study. Osteoarthritis Cartilage 12: 46-55.

40. Mazzuca SA, Page MC, Meldrum RD, Brandt KD, Petty-Saphon S (2004) Pilot study of the effects of a heat-retaining knee sleeve on joint pain, stiffness, and function in patients with knee osteoarthritis. Arthritis Rheum 51: 716-721.

41. Nuhr M, Hoerauf K, Bertalanffy A, Bertalanffy P, Frickey N, et al. (2004) Active warming during emergency transport relieves acute low back pain. Spine 29: 1499-1503.

42. Colford JM, Jr., Rees JR, Wade TJ, Khalakdina A, Hilton JF, et al. (2002) Participant blinding and gastrointestinal illness in a randomized, controlled trial of an in-home drinking water intervention. Emerg Infect Dis 8: 29-36.

43. Terreehorst I, Hak E, Oosting AJ, Tempels-Pavlica Z, de Monchy JG, et al. (2003) Evaluation of impermeable covers for bedding in patients with allergic rhinitis. N Engl J Med 349: 237-246.

44. Felson DT, LaValley MP, Baldassare AR, Block JA, Caldwell JR, et al. (1999) The Prosorba column for treatment of refractory rheumatoid arthritis: a randomized, double-blind, sham-controlled trial. Arthritis Rheum 42: 2153-2159.

45. Myles PS, Leslie K, McNeil J, Forbes A, Chan MT (2004) Bispectral index monitoring to prevent awareness during anaesthesia: the B-Aware randomised controlled trial. Lancet 363: 1757-1763.

46. Michlovitz S, Hun L, Erasala GN, Hengehold DA, Weingand KW (2004) Continuous low-level heat wrap therapy is effective for treating wrist pain. Arch Phys Med Rehabil 85: 1409-1416.

47. Waksman R, Ajani AE, White RL, Chan R, Bass B, et al. (2004) Five-year follow-up after intracoronary gamma radiation therapy for in-stent restenosis. Circulation 109: 340-344.

48. Bradley JD, Heilman DK, Katz BP, Gsell P, Wallick JE, et al. (2002) Tidal irrigation as treatment for knee osteoarthritis: a sham- controlled, randomized, double-blinded evaluation. Arthritis Rheum 46: 100-108.

49. Freed CR, Greene PE, Breeze RE, Tsai WY, DuMouchel W, et al. (2001) Transplantation of embryonic dopamine neurons for severe Parkinson's disease. N Engl J Med 344: 710-719.

50. Geurts JW, van Wijk RM, Wynne HJ, Hammink E, Buskens E, et al. (2003) Radiofrequency lesioning of dorsal root ganglia for chronic lumbosacral radicular pain: a randomised, double-blind, controlled trial. Lancet 361: 21-26.

51. Moseley JB, O'Malley K, Petersen NJ, Menke TJ, Brody BA, et al. (2002) A controlled trial of arthroscopic surgery for osteoarthritis of the knee. N Engl J Med 347: 81-88.

52. Sung JJ, Chan FK, Lau JY, Yung MY, Leung WK, et al. (2003) The effect of endoscopic therapy in patients receiving omeprazole for bleeding ulcers with nonbleeding visible vessels or adherent clots: a randomized comparison. Ann Intern Med 139: 237-243.

53. Swank DJ, Swank-Bordewijk SC, Hop WC, van Erp WF, Janssen IM, et al. (2003) Laparoscopic adhesiolysis in patients with chronic abdominal pain: a blinded randomised controlled multi-centre trial. Lancet 361: 1247-1251.

54. Buchbinder R, Green S, Forbes A, Hall S, Lawler G (2004) Arthrographic joint distension with saline and steroid improves function and reduces pain in patients with painful stiff shoulder: results of a randomised, double blind, placebo controlled trial. Ann Rheum Dis 63: 302-309.

55. van Geel N, Ongenae K, De Mil M, Haeghen YV, Vervaet C, et al. (2004) Double-blind placebo-controlled study of autologous transplanted epidermal cell suspensions for repigmenting vitiligo. Arch Dermatol 140: 1203-1208.

56. Chang AK, Schoeman G, Hill M (2004) A randomized clinical trial to assess the efficacy of the Epley maneuver in the treatment of acute benign positional vertigo. Acad Emerg Med 11: 918-924.

57. Balon J, Aker PD, Crowther ER, Danielson C, Cox PG, et al. (1998) A comparison of active and simulated chiropractic manipulation as adjunctive treatment for childhood asthma. N Engl J Med 339: 1013-1020.

58. Bennell KL, Hinman RS, Metcalf BR, Buchbinder R, McConnell J, et al. (2005) Efficacy of physiotherapy management of knee joint osteoarthritis: a randomised, double blind, placebo controlled trial. Ann Rheum Dis 64: 906-912.

59. Baker KR, Nelson ME, Felson DT, Layne JE, Sarno R, et al. (2001) The efficacy of home based progressive strength training in older adults with knee osteoarthritis: a randomized controlled trial. J Rheumatol 28: 1655-1665.

60. Atkinson W, Sheldon TA, Shaath N, Whorwell PJ (2004) Food elimination based on IgG antibodies in irritable bowel syndrome: a randomised controlled trial. Gut 53: 1459-1464.

61. Hogberg L, Laurin P, Falth-Magnusson K, Grant C, Grodzinsky E, et al. (2004) Oats to children with newly diagnosed coeliac disease: a randomised double blind study. Gut 53: 649-654.

62. Bertalanffy P, Hoerauf K, Fleischhackl R, Strasser H, Wicke F, et al. (2004) Korean hand acupressure for motion sickness in prehospital trauma care: a prospective, randomized, double-blinded trial in a geriatric population. Anesth Analg 98: 220-223, table of contents.

63. Brygge T, Heinig JH, Collins P, Ronborg S, Gehrchen PM, et al. (2001) Reflexology and bronchial asthma. Respir Med 95: 173-179.

64. Edinger JD, Wohlgemuth WK, Radtke RA, Marsh GR, Quillian RE (2001) Cognitive behavioral therapy for treatment of chronic primary insomnia: a randomized controlled trial. Jama 285: 1856-1864.

65. Edworthy SM, Devins GM (1999) Improving medication adherence through patient education distinguishing between appropriate and inappropriate utilization. Patient Education Study Group. J Rheumatol 26: 1793-1801.

66. Koes BW, Bouter LM, van Mameren H, Essers AH, Verstegen GM, et al. (1992) The effectiveness of manual therapy, physiotherapy, and treatment by the general practitioner for nonspecific back and neck complaints. A randomized clinical trial. Spine 17: 28-35.

67. Petrella RJ, Bartha C (2000) Home based exercise therapy for older patients with knee osteoarthritis: a randomized clinical trial. J Rheumatol 27: 2215-2221.

68. Jang HS, Lee MS (2004) Effects of qi therapy (external qigong ) on premenstrual syndrome: a randomized placebo-controlled study. J Altern Complement Med 10: 456-462.

69. Lewis AK, Osborn IP, Roth R (2004) The effect of hemispheric synchronization on intraoperative analgesia. Anesth Analg 98: 533-536, table of contents.

70. Rubens FD, Dupuis JY, Robblee J, Rock G, Bryson GL, et al. (2000) Feasibility of blinding in a randomized controlled trial comparing preoperative autologous blood donation and acute normovolemic hemodilution in adult cardiac surgery. Transfusion 40: 1058-1062.

71. Annane D, Depondt J, Aubert P, Villart M, Gehanno P, et al. (2004) Hyperbaric oxygen therapy for radionecrosis of the jaw: a randomized, placebo-controlled, double-blind trial from the ORN96 study group. J Clin Oncol 22: 4893-4900.

72. Scavone BM, Wong CA, Sullivan JT, Yaghmour E, Sherwani SS, et al. (2004) Efficacy of a prophylactic epidural blood patch in preventing post dural puncture headache in parturients after inadvertent dural puncture. Anesthesiology 101: 1422-1427.
