# Supplementary material for: Reporting Methods of Blinding in Randomized Trials Assessing Nonpharmacological Treatments
Source: PLoS Med. 2007 Feb 20;4(2):e61. doi: 10.1371/journal.pmed.0040061 (PMC1800311; doi:10.1371/journal.pmed.0040061)
Supplement: Supplementary file 3 [file pmed.0040061.st002.doc]

Table S2: Methods of blinding patients to study hypothesis

| **Category of treatment assessed** | **Methods of blinding** |
| --- | --- |
| Devices |  |
| Parental auricular acupuncture as an adjunct for parental presence during induction of anesthesia^1^ | The research assistant informed patients that they might or not experience a pinching sensation associated with insertion of the needles. They were informed that this sensation was not related to group assignment. |
| Can a standardized acupuncture technique palliate disabling breathlessness? A single-blind, placebo-controlled crossover study^2^ (316) | In the control arm patients were instructed that the treatment was high –frequency and low intensity stimulation and that they would probably feel no sensation.  Treatment credibility was assessed. |
| Acupuncture versus placebo for the treatment of chronic mechanical neck pain: a randomized, controlled trial^3^ | Same as above (partial information of specific characteristics of the treatments in each arm) |
| The comparative analgesic efficacy of transcutaneous electrical nerve stimulation and a non-steroidal anti-inflammatory drug for painful osteoarthritis^4^ | Same as above (partial information of specific characteristics of the treatments in each arm) |
| Sham radiation in clinical trials assessing radiotherapy for exudative age-related macular degeneration^5^ | Same as above (partial information of specific characteristics of the treatments in each arm) |
| Constant vs auto-continuous positive airway pressure in patients with sleep apnea hypopnea syndrome and a high variability in pressure requirement^6^ | Subjects were told that they would sleep with a machine functioning in two distinct modes. No further explanation was given and specific questions were not answered. |
| Laterally elevated wedged insoles in the treatment of medial knee osteoarthritis. A two-year prospective randomized controlled study^7^ | Patients were told that the study aimed to compare two kinds of insoles, but not that one was presumed to be of greater efficacy than the other. |
| Taping the patella medially: a new treatment for osteoarthritis of the knee joint?^8^ | Same as above (blind to study hypothesis, i.e., not informing that one treatment was presumed to be of greater efficacy than the other or was a placebo) |
| Percutaneous electrical nerve stimulation for low back pain: a randomized crossover study^9^ | Same as above (blind to study hypothesis) |
| Effect of firmness of mattress on chronic non-specific low-back pain: randomised, double-blind, controlled, multicentre trial^10^ | Same as above (blind to study hypothesis) |
| The placebo needle, is it a valid and convincing placebo for use in acupuncture trials? A randomised, single-blind, cross-over pilot trial^11^ | Same as above (blind to study hypothesis) |
|  |  |
| Rehabilitation / diet |  |
| Home based progressive strength training for osteoarthritis^12^ | Patients were blinded to the active treatment.  Although nutrition was not the active intervention, patients were informed that investigators were examining the effects of both exercises and nutrition. |
| Manual therapy, physiotherapy for back and neck pain^13^ | Patients were blind to the placebo therapy. |
| Repetitive bilateral arm training and motor cortex activation in chronic stroke^14^ | Patients were aware of the differences in treatments but were not specifically aware that one treatment was a control because neither the consent forms nor verbal explanations referred to DMTE as a control treatment. Thus patients could reasonably expect an improvement regardless of treatment group. |
| Physiotherapy, including quadriceps exercises and patellar taping, for knee osteoarthritis with predominant patello-femoral joint involvement: randomized controlled trial^15^ | A modified Zelen design to blind patients to the study hypothesis was reported in a trial comparing usual care with a complex, physical therapy-based intervention for patello-femoral joint osteoarthritis of the knee. At the first stage, patients were invited to participate in a cohort. At the second stage, patients randomized to the intervention arm were informed that they would receive the experimental treatment and signed a second consent form. |
| Knee tape for osteoarthritis^16^ | 3 arms: active tape, control tape, no tape. Patients were unaware of which taping technique was considered therapeutic. |
| Cognitive behavioral therapy for treatment for chronic primary insomnia^17^ (A40) | Enrollees were blinded to the hypotheses and nature of the placebo therapy but were told they had a 1 in 3 chance of placebo treatment.  Therapists were blinded to hypothesis and were uninformed that 1 of the treatments they administered was a placebo. Enrollees were instructed not to inform their therapists about the placebo condition.  Therapists were beginning-level psychologists, naïve to behavioral insomnia therapy. Once all enrollees completed treatment, therapists were debriefed about the study hypothesis and placebo treatment.  Treatment credibility was assessed. |
| Other treatments |  |
| Effects of Qi Therapy on premenstrual syndrome^18^ | The subjects were informed that they would receive one of two types of Qi therapy (the Qi master concentrates on the area of perceived accumulated tension in the subject’s body and subjectively “projects “ qi from his hand), each of which had potential to relieve premenstrual symptoms. They did not know which treatment they received, real or sham qi therapy. In the sham Qi therapy, the mimic was similar but without any effort or intention to emit real Qi. |

**References**

1. Wang SM, Maranets I, Weinberg ME, Caldwell-Andrews AA, Kain ZN. Parental auricular acupuncture as an adjunct for parental presence during induction of anesthesia. Anesthesiology 2004;100(6):1399-404.

2. Lewith GT, Prescott P, Davis CL. Can a standardized acupuncture technique palliate disabling breathlessness: a single-blind, placebo-controlled crossover study. Chest 2004;125(5):1783-90.

3. White P, Lewith G, Prescott P, Conway J. Acupuncture versus placebo for the treatment of chronic mechanical neck pain: a randomized, controlled trial. Ann Intern Med 2004;141(12):911-9.

4. Lewis B, Lewis D, Cumming G. The comparative analgesic efficacy of transcutaneous electrical nerve stimulation and a non-steroidal anti-inflammatory drug for painful osteoarthritis. Br J Rheumatol 1994;33(5):455-60.

5. Marcus DM, Camp MW, Sheils WC, McIntosh SB, Leibach DB, Johnson MH, et al. Sham radiation in clinical trials assessing radiotherapy for exudative age-related macular degeneration. Retina 1999;19(6):525-30.

6. Noseda A, Kempenaers C, Kerkhofs M, Braun S, Linkowski P, Jann E. Constant vs auto-continuous positive airway pressure in patients with sleep apnea hypopnea syndrome and a high variability in pressure requirement. Chest 2004;126(1):31-7.

7. Pham T, Maillefert JF, Hudry C, Kieffert P, Bourgeois P, Lechevalier D, et al. Laterally elevated wedged insoles in the treatment of medial knee osteoarthritis. A two-year prospective randomized controlled study. Osteoarthritis Cartilage 2004;12(1):46-55.

8. Cushnaghan J, McCarthy C, Dieppe P. Taping the patella medially: a new treatment for osteoarthritis of the knee joint? Bmj 1994;308(6931):753-5.

9. Ghoname EA, Craig WF, White PF, Ahmed HE, Hamza MA, Henderson BN, et al. Percutaneous electrical nerve stimulation for low back pain: a randomized crossover study. Jama 1999;281(9):818-23.

10. Kovacs FM, Abraira V, Pena A, Martin-Rodriguez JG, Sanchez-Vera M, Ferrer E, et al. Effect of firmness of mattress on chronic non-specific low-back pain: randomised, double-blind, controlled, multicentre trial. Lancet 2003;362(9396):1599-604.

11. White P, Lewith G, Hopwood V, Prescott P. The placebo needle, is it a valid and convincing placebo for use in acupuncture trials? A randomised, single-blind, cross-over pilot trial. Pain 2003;106(3):401-409.

12. Baker KR, Nelson ME, Felson DT, Layne JE, Sarno R, Roubenoff R. The efficacy of home based progressive strength training in older adults with knee osteoarthritis: a randomized controlled trial. J Rheumatol 2001;28(7):1655-65.

13. Koes BW, Bouter LM, van Mameren H, Essers AH, Verstegen GM, Hofhuizen DM, et al. The effectiveness of manual therapy, physiotherapy, and treatment by the general practitioner for nonspecific back and neck complaints. A randomized clinical trial. Spine 1992;17(1):28-35.

14. Luft AR, McCombe-Waller S, Whitall J, Forrester LW, Macko R, Sorkin JD, et al. Repetitive bilateral arm training and motor cortex activation in chronic stroke: a randomized controlled trial. Jama 2004;292(15):1853-61.

15. Quilty B, Tucker M, Campbell R, Dieppe P. Physiotherapy, including quadriceps exercises and patellar taping, for knee osteoarthritis with predominant patello-femoral joint involvement: randomized controlled trial. J Rheumatol 2003;30(6):1311-7.

16. Hinman RS, Crossley KM, McConnell J, Bennell KL. Efficacy of knee tape in the management of osteoarthritis of the knee: blinded randomised controlled trial. Bmj 2003;327(7407):135.

17. Edinger JD, Wohlgemuth WK, Radtke RA, Marsh GR, Quillian RE. Cognitive behavioral therapy for treatment of chronic primary insomnia: a randomized controlled trial. Jama 2001;285(14):1856-64.

18. Jang HS, Lee MS. Effects of qi therapy (external qigong ) on premenstrual syndrome: a randomized placebo-controlled study. J Altern Complement Med 2004;10(3):456-62.
