# Supplementary material for: Reporting Methods of Blinding in Randomized Trials Assessing Nonpharmacological Treatments
Source: PLoS Med. 2007 Feb 20;4(2):e61. doi: 10.1371/journal.pmed.0040061 (PMC1800311; doi:10.1371/journal.pmed.0040061)
Supplement: Supplementary file 4 [file pmed.0040061.st003.doc]

Table S3: Methods to blind outcome assessors

|  | **Methods of blinding** |
| --- | --- |
| Physician-driven data |  |
| Role of first ray hypermobility in the outcome of the Hohmann and the Lapidus procedure. A prospective, randomized trial involving one hundred and one feet ^1^ | Tape was applied to the dorsal and medial sides of the foot by the nurse to hide the surgical scars so that examiners was blinded to the procedure that had been performed |
| Unilateral pallidotomy versus bilateral subthalamic nucleus stimulation in PD: a randomized trial^2^ | Operation scars were masked by a head cap. |
| Randomized trial of pallidotomy versus medical therapy for Parkinson's disease^3^ | Patients wore hats to mask the scars and evaluating neurologists had no contact with the patient between visits and no involvement in their ongoing care. |
| Interactive music therapy as a treatment for preoperative anxiety in children^4^ | Assessors were trained by using videotapes of children undergoing the induction of anesthesia to reliably measure children’s anxiety with the Yale Preoperative Anxiety Scale. Assessors were kept blinded to the purpose of the study and the group involved. All children in the study were videotaped in the holding area until the induction of anesthesia. The blinded assessors then used the videotapes to rate children’s anxiety for this study. The music therapist was visible on video. |
| Analgesic effect of breast feeding in term neonates: randomised controlled trial^5^ | Participating infants and their mothers were taken to a quiet nursery room for venepuncture. One of the researchers opened a consecutively numbered envelope, which contained the treatment assigned to each infant. Infants were allocated to one of four groups: in group 1 they were breast fed, starting 2 min before the procedure and continuing throughout; in group 2 they were held in their mother's arms without breast feeding, starting 2 min before the procedure; in group 3, infants were laid on a table and given 1 ml of placebo (sterile water) without a pacifier 2 min before the procedure; and in group 4  infants were laid on a table and given 1 ml of 30% glucose followed by sucking a pacifier 2 min before the procedure. The infant's legs and feet were uncovered to allow observation of movements. Infants in groups 3 and 4 lay supine on an examination table during procedure. The infants and the monitor screen were video recorded during the procedure. When all inclusions were completed, two specially trained observers independently assessed the recordings using the Douleur Aiguë Nouveau-né (DAN) scale (primary outcome measure) and the premature infant pain profile (PIPP) scale (secondary outcome measure). Observers could stop and restart the videotape as many times as they needed to establish a score. These observers were blinded to the purpose and hypothesis of the study. They had been told that investigators were assessing agreement of their scores in four different situations. |
| Surgery for idiopathic full-thickness macular hole: two-year results of a randomized clinical trial comparing natural history, vitrectomy, and vitrectomy plus autologous serum: ^6^ | Photographic and fundus fluorecein angiographic assessments were performed by examining digitalized images, which were coded separately so that the assessor was masked to the identity of the patient. |
| Efficacy of multiwavelength light therapy in the treatment of pressure ulcers in subjects with disorders of the spinal cord^7^ | Photographs of the ulcers were taken at the beginning of the treatment, end of treatment and 14 days after the last session. All evaluations were done by a blinded outcome assessor. |
| Treatment of acne vulgaris with a pulsed dye laser: a randomized controlled trial^8^ | Standardized bilateral facial photographs were obtained at baseline and every 2 weeks throughout the study. Participants were photographed at a fixed distance with use of a Nikon D1x digital camera (Nikon Corp, Tokyo, Japan). Participants had been positioned at a defined spot in the studio and instructed to look at a fixed target during photography. Lighting was provided by studio strobe lights fixed in position, and images were exposed at f22. Views included face front and left and right profiles. Images were taken at a reproduction ratio equivalent of 1:6 on 35-mm film. Subsequently, participants' photographs were viewed by a panel of 3 dermatologists who were blinded to treatment side. The severity of acne in the images obtained at baseline, week 4, and week 12 was then graded using the Leeds acne severity scale, an ordinal photonumeric scale ranging from 1 (least severe) to 12 (most severe), for both treated and untreated sides of the face. |
| Effects of semantic treatment on verbal communication and linguistic processing in aphasia after stroke^9^ | The primary outcome measure was a valid and reliable measure of verbal communicative ability. Patient responses were tape recorded and scored by 2 independent blinded observers. |
| Suturing versus conservative management of lacerations of the hand^10^ | At 3-month follow up, the research assistant took a digital photograph of patients' healed wounds. Two independent doctors, who were unaware of the method of treatment, rated the photographs for cosmetic appearance on a previously validated visual analogue scale. This scale is 100-mm long, with "worst scar" 0 mm and "best scar" 100 mm. A difference of 12-15 mm is clinically important. Patients rated their own scars on a similar scale |
| Complementary test |  |
| Off-pump vs conventional coronary artery bypass grafting: early and 1-year graft patency, cost, and quality-of-life outcomes: a randomized trial^11^ | 3 cardiologists who were blinded to group assignment simultaneously reviewed angiograms. |
| Event |  |
| Fall prevention in residential care: a cluster, randomized, controlled trial^12^ | Fall questionnaires were completed by care providers and other caregivers and were assessed by trained blinded outcome assessors to determine whether it was a fall or not. Quality control procedures on the questionnaire and the main document were provided. |
| Public-access defibrillation and survival after out-of-hospital cardiac arrest^13^ | An event adjudication committee classified all presumed cardiac arrests and was blinded to treatment allocation. The committee received a a masked narrative report of each event with use of sham masking blacking out or altering reports to disguise whether the device was used or not. |
| The Lambeth Early Onset (LEO) Team: randomised controlled trial of the effectiveness of specialised care for early psychosis^14^ | 2 blinded researchers rated recovery and relapse using operationalized criteria applied to extracts of the clinical case notes from which information about group allocation was removed. |
| **Multiple outcome or composite outcome** | |
| Effectiveness of exercise in patients with osteoarthritis of hip or knee: nine months' follow up^15^ | Observed disability was determined by studying videos of the patients’ performance of a series of standardized tasks. |
| Clinical assessment of pediatric obstructive sleep apnea^16^ | Audiotape recording the child’s breathing while asleep (sleep tape). At least 20 min of the tape was evaluated by the principal investigator and scored. |

**References**

1. Faber FW, Mulder PG, Verhaar JA. Role of first ray hypermobility in the outcome of the Hohmann and the Lapidus procedure. A prospective, randomized trial involving one hundred and one feet. J Bone Joint Surg Am 2004;86-A(3):486-95.

2. Esselink RA, de Bie RM, de Haan RJ, Lenders MW, Nijssen PC, Staal MJ, et al. Unilateral pallidotomy versus bilateral subthalamic nucleus stimulation in PD: a randomized trial. Neurology 2004;62(2):201-7.

3. Vitek JL, Bakay RA, Freeman A, Evatt M, Green J, McDonald W, et al. Randomized trial of pallidotomy versus medical therapy for Parkinson's disease. Ann Neurol 2003;53(5):558-69.

4. Kain ZN, Caldwell-Andrews AA, Krivutza DM, Weinberg ME, Gaal D, Wang SM, et al. Interactive music therapy as a treatment for preoperative anxiety in children: a randomized controlled trial. Anesth Analg 2004;98(5):1260-6, table of contents.

5. Carbajal R, Veerapen S, Couderc S, Jugie M, Ville Y. Analgesic effect of breast feeding in term neonates: randomised controlled trial. Bmj 2003;326(7379):13.

6. Ezra E, Gregor ZJ. Surgery for idiopathic full-thickness macular hole: two-year results of a randomized clinical trial comparing natural history, vitrectomy, and vitrectomy plus autologous serum: Morfields Macular Hole Study Group RAeport no. 1. Arch Ophthalmol 2004;122(2):224-36.

7. Taly AB, Sivaraman Nair KP, Murali T, John A. Efficacy of multiwavelength light therapy in the treatment of pressure ulcers in subjects with disorders of the spinal cord: A randomized double-blind controlled trial. Arch Phys Med Rehabil 2004;85(10):1657-61.

8. Orringer JS, Kang S, Hamilton T, Schumacher W, Cho S, Hammerberg C, et al. Treatment of acne vulgaris with a pulsed dye laser: a randomized controlled trial. Jama 2004;291(23):2834-9.

9. Doesborgh SJ, van de Sandt-Koenderman MW, Dippel DW, van Harskamp F, Koudstaal PJ, Visch-Brink EG. Effects of semantic treatment on verbal communication and linguistic processing in aphasia after stroke: a randomized controlled trial. Stroke 2004;35(1):141-6.

10. Quinn J, Cummings S, Callaham M, Sellers K. Suturing versus conservative management of lacerations of the hand: randomised controlled trial. Bmj 2002;325(7359):299.

11. Puskas JD, Williams WH, Mahoney EM, Huber PR, Block PC, Duke PG, et al. Off-pump vs conventional coronary artery bypass grafting: early and 1-year graft patency, cost, and quality-of-life outcomes: a randomized trial. Jama 2004;291(15):1841-9.

12. Kerse N, Butler M, Robinson E, Todd M. Fall prevention in residential care: a cluster, randomized, controlled trial. J Am Geriatr Soc 2004;52(4):524-31.

13. Hallstrom AP, Ornato JP, Weisfeldt M, Travers A, Christenson J, McBurnie MA, et al. Public-access defibrillation and survival after out-of-hospital cardiac arrest. N Engl J Med 2004;351(7):637-46.

14. Craig TK, Garety P, Power P, Rahaman N, Colbert S, Fornells-Ambrojo M, et al. The Lambeth Early Onset (LEO) Team: randomised controlled trial of the effectiveness of specialised care for early psychosis. Bmj 2004;329(7474):1067.

15. van Baar ME, Dekker J, Oostendorp RA, Bijl D, Voorn TB, Bijlsma JW. Effectiveness of exercise in patients with osteoarthritis of hip or knee: nine months' follow up. Ann Rheum Dis 2001;60(12):1123-30.

16. Goldstein NA, Pugazhendhi V, Rao SM, Weedon J, Campbell TF, Goldman AC, et al. Clinical assessment of pediatric obstructive sleep apnea. Pediatrics 2004;114(1):33-43.
